# Superficial layer pyramidal cells communicate heterogeneously between multiple functional domains of cat primary visual cortex

**DOI:** 10.1038/ncomms6252

**Published:** 2014-10-24

**Authors:** Kevan A. C. Martin, Stephan Roth, Elisha S. Rusch

**Affiliations:** 1 Institute of Neuroinformatics, UZH/ETH, Winterthurerstrasse 190, 8057 Zürich, Switzerland

## Abstract

The axons of pyramidal neurons in the superficial layers of the neocortex of higher mammals form lateral networks of discrete clusters of synaptic boutons. In primary visual cortex the clusters are reported to link domains that share the same orientation preferences, but how individual neurons contribute to this network is unknown. Here we performed optical imaging to record the intrinsic signal, which is an indirect measure of neuronal firing, and determined the global map of orientation preferences in the cat primary visual system. In the same experiment, single cells were recorded and labelled intracellularly. We found that individual axons arborise within the retinotopic representation of the classical receptive field, but their bouton clusters were not aligned along their preferred axis of orientation along the retinotopic map. Axon clusters formed in a variety of different orientation domains, not just the like-orientation domains. This topography and heterogeneity of single-cell connectivity provides circuits for normalization and context-dependent feature processing of visual scenes.

## Abstract (web summary)

Pyramidal cells in superficial layers of neocortex form a lateral network of synaptic bouton clusters, but its functional implication remains unclear. Here the authors overlaid activity maps of orientation preference in cat visual cortex with single-cell anatomy and observed projections to multiple functional domains.

## Graphical abstract

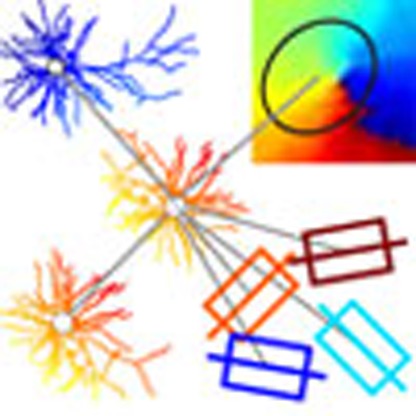

The neocortex is thought to be constructed of a system of radial ‘columns’ that form the functional units of cortex^1^. In higher mammals these columns are interlinked by lateral excitatory connections that form a patchy network known as the cortical ‘daisy’^2^,^3^. Over the last few decades, many attempts have been made to decipher the significance of the daisy using the primary visual cortex (V1) as the test example. The current consensus is the lateral excitatory connections extend along the retinotopic axis of the preferred orientation where they link columns that share the same orientation preferences, that is, expressed as ‘like-to-like’ connectivity.

The regular periodic representation of orientation selectivity is the most dominantly expressed functional map in V1 of many species. The ‘like-to-like’ pattern of underlying connections^4^,^5^,^6^,^7^ are thought to be a natural consequence of a Hebbian ‘fire-together-wire-together’ mechanism. However, there are major conceptual problems with this explanation. First, the correct stimulus orientation is clearly essential to drive a neuron well, but who actually fires together during natural stimulation is a far more complex affair given there are other spatio-temporal parameters strongly influencing the response (for example, direction of motion, spatial and temporal frequency and contrast sensitivity). Second, the correlation between anatomical patches and the global orientation map was derived from a relatively small number of extracellular injections of tracers that label many neurons. That method hides significant details in the connection patterns of single neurons, so we do not know whether the poor precision of the ‘like-to-like’ wiring is a bug or a feature. Finally, the pyramidal cells in the superficial layers of cat V1 are more richly recurrently connected with each other than neurons in any other layer^8^, and so ‘like-to-like’ would generate a hazardous positive feedback unless it were balanced with strong inhibition^9^, which seems not to be present.

To circumvent these interpretive obstacles and arrive at a view at single-cell resolution, we imaged an optical population signal, the so-called ‘intrinsic signal’, to recover the global orientation map in the cat primary visual cortex and combined this with single-cell physiology and intracellular labelling *in vivo*. After fine-grained three-dimensional (3D) reconstruction of 33 neurons, we found that all neurons formed anisotropic lateral projections that covered only a few degrees of visual field representation on the cortex, matching the size of the classical receptive field (RF). These anisotropies were unrelated to the orientation preference of the parent neuron, or the cardinal axes of the retinotopic map. We calculated a ‘Similarity Index’ (SI) to compare the orientation domains innervated by each synaptic bouton cluster with the domains occupied by the parent cells dendrite. Our analyses gave SI values ranging from 0.13 to 0.96 (1.0 indicated most similar, 0 least similar). This indicates that individual pyramidal cells project laterally to multiple orientation domains, not simply like-to-like. Hence, superficial pyramidal cells form a highly structured, but heterogeneous network, which seems organized to ensure to optimize the combinations of contextual cues that can influence the processing of objects in natural scenes.

## Results

### Neurons and their clusters

**Figure 1:**
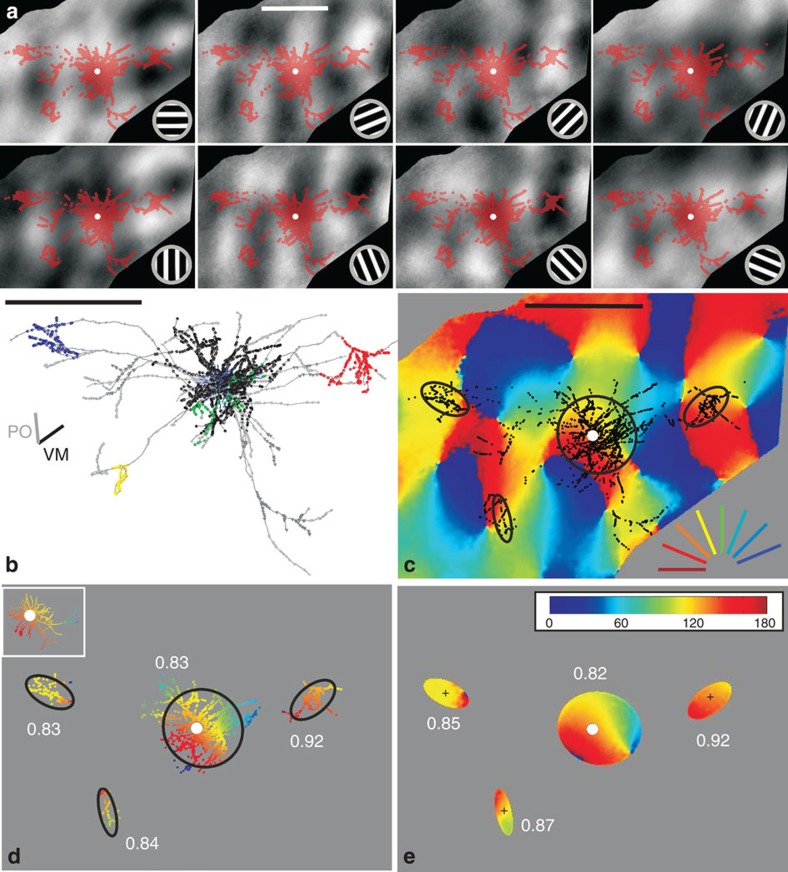
Synaptic boutons of one typical superficial layer pyramidal neuron overlaid on maps of cortical activity. (**a**) Activity response maps (grey scale images) in response to moving gratings at orientations indicated at the bottom right. Darker pixels indicate stronger responses. Overlaid are synaptic boutons (red dots) from the neuron in **b** (soma=white dot). (**b**) Top-view of a typical superficial layer pyramidal neuron. Neuron’s axonal tree in grey, individual bouton cluster highlighted with coloured dots and the dendritic tree in blue. The bars left denote the vertical meridian (‘VM’) and neuron’s preferred orientation (‘PO’) (for more details see Supplementary Fig. 1). (**c**) The eight greyscale maps from **a** were used to generate the conventional colour-coded orientation map (orientation indicated at the bottom right) (see Methods). Overlaid are the superficial layer synaptic boutons in black (white dot=soma). 2D ellipses were fitted to each individual bouton cluster (black curves). (**d**) Only boutons belonging to clusters, and to the dendritic tree (inset), were colour coded by their corresponding pixel value of the map, the rest faded in grey. The similarity between a neuron’s dendritic tree and its bouton clusters was expressed with a ‘Similarity Index’ (SI), see white numbers in **d** and **e**. SI of 1 means a perfect match, 0 means complete mismatch (that is, a 90-degree difference see Fig. 2 and Methods). (**e**) Only regions within the fitted ellipses (black ellipses in **c**) were considered for calculating the SI. Note that bouton clusters (in **d**) and their fitted ellipses (in **e**) have very similar SIs. (**a**–**e**) Scale bars, 1 mm. (Neuron ID: 11, cat_1007_RH_neuron_01. Receptive field properties: complex, ocular dominance 4, size 1 × 1°, location −1°/−3.6° from area centralis, preferred orientation 150°±9°, direction selective).

Our analyses are based on full 3D reconstructions of 33 intracellularly filled neurons from 15 hemispheres of 13 cats. Of 153 impaled neurons 50 were ‘best-fills’ (see Methods), but after excluding neurons associated with indifferent optical imaging maps, we arrived at a final data set of 33 neurons (see each of them in Supplementary Figs 1–4). Instead of the customary subjective delineation of a bouton cluster, we used a mean-shift algorithm^10^, which we developed previously to give a more objective basis for identifying the clusters in 3D (see Methods). Figure 1 indicates in colour the boutons that lay in clusters identified by the mean-shift algorithm (for example, Fig. 1b). Boutons that lay along unbranched segments of the axon, or which were not part of an identified cluster, were excluded from further analyses. In five neurons the algorithm detected only one superficial cluster (see all 33 neurons in Supplementary Figs 3 and 4). For simplification, the 3D bouton cluster was parameterized in 3D by fitting an ellipsoid and by an ellipse in two-dimensional (2D) projections (see Methods).

### Functional maps

To determine the organization of orientation preferences over several sq. mm of V1, we recorded the cortical responses to oriented moving sinewave gratings by wide-field imaging of the ‘intrinsic signal’. This intrinsic signal is measured by recording the changes in cortical reflectance linked to activity-dependent changes in blood flow, oxy/deoxy haemaglobin ratio and tissue light scattering (see Fig. 1a). Although not able to provide single-cell resolution, intrinsic imaging is particularly well suited for functional mapping of the larger-scale columnar systems such as orientation or ocular dominance. Here we used the ‘orientation map’ as the basis for determining the specificity of inter-orientation domain wiring by individual neurons.

### Functional specificity of bouton clusters

**Figure 2:**
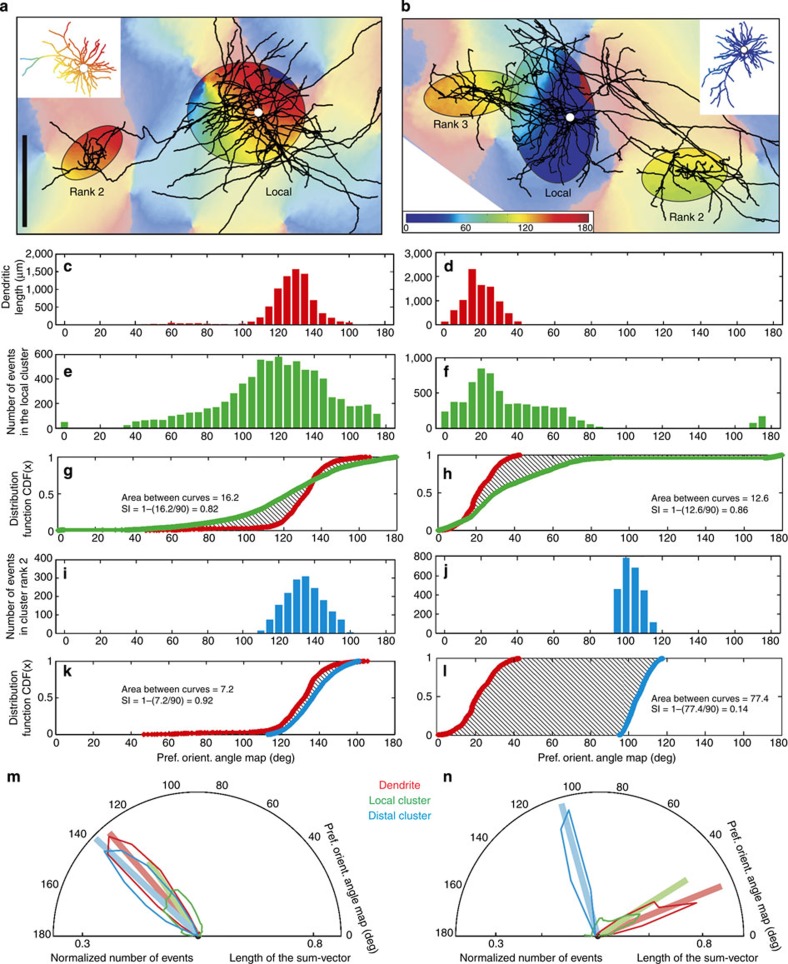
Calculation of the Similarity Index (SI) with the cumulative distribution function (CDF)-method exemplified with two contrasting neurons (left and right columns). (**a**,**b**) Axon and orientation map. For clarity, boutons were removed and only ellipses shown for the local cluster (rank 1) and the superficial layer clusters (ranks indicated). Dendritic trees (insets) were colour coded by the orientation value of their corresponding pixels (soma=white dot). Scale bar, 0.5 mm. (**c**,**d**) Histogram of the pixel’s orientation values underlying the dendritic tree (insets in **a** and **b**). (**e**,**f**) Histogram of orientation pixel values for the region enclosed by a local cluster ellipse. (**i**,**j**) Equivalent histogram for one distal ellipse (rank 2). (**g**,**h**) CDF for dendrites (red) and local ellipse (green). Area differences between CDFs were used to calculate the SI (see text in graph). (**k**,**l**) CDF for dendrites and rank 2 distal cluster (blue). The formula for the SI is given in the graph. (**m**,**n**) Hemispheric plots were created by converting the histograms into radial values, normalized by the total number of events (coloured curves). The individual vectors forming these hemispheric plots were summed up to generate one sum-vector (bold vector). Supplementary Fig. 5 shows the sum-vectors for each of the 33 neurons. The length of this sum-vector is termed as the ‘tuning’ of the dendrite or cluster. (Neuron ID: **a** (11), see Fig. 1; **b** (17), cat_0608_RH_neuron_01. Receptive field properties: complex, ocular dominance 1, size 1.4 × 2.7°, location 0.5°/−2° from area centralis, preferred orientation 63°±47°, direction preference 153°).

Figure 2a,b shows two example neurons selected because of their very different distal projections in relation to the orientation map. The relationship of anatomy to functional map was quantified by comparing the orientation preferences of the domains innervated by individual bouton clusters with the domains occupied by the dendritic tree by means of a ‘SI’. To do so, we assigned to each 1-micron segment of the dendritic tree the mean preferred orientation of the underlying pixels (insets Fig. 2a,b) and we determined the range of orientations contained within the ellipse fitted to the bouton clusters (Fig. 2a,b). The SI was then derived from the difference between the cumulative distribution functions (CDFs) of the dendrites and the cluster (see Methods). An SI of 1 indicates a perfect match between dendrites and axon, whereas 0 indicates a 90-degree mismatch, that is, a bouton cluster formed at the orthogonal orientation. Figure 2 exemplifies the calculation of the SI values. Each dendritic segment and each bouton belonging to a cluster was superimposed on the orientation map and assigned an orientation value based on the pixel value for that location on the map. The results were histogrammed for the dendritic segments (Fig. 2c,d) and similar histograms were constructed for the pixel values enclosed by the ellipses representing local axon clusters (Fig. 2e,f) and the rank 2 distal cluster (Fig. 2i,j). These pixel values were expressed as a CDF, which compares the dendrites with the local cluster (for example, Fig. 2g,h) and with the distal clusters (Fig. 2k,l). Then, based on the CDF, the SIs were calculated. In this instance, both the local and the largest distal cluster (rank 2) of the neuron shown in Fig. 2a gave high SIs (0.82, 0.92, respectively), indicating that they sampled from similar regions of the orientation map as the dendritic tree. However, for the second neuron (Fig. 2b), the local cluster had an SI of 0.86, but the largest distal cluster (rank 2) had an SI of 0.14, indicating it occupied a very different set of orientation preferences compared with its dendritic tree.

### Orientation tuning

The orientation ‘tuning’ of the dendrites and clusters was assessed by converting the histogram of the orientation values at each bouton cluster into a half-polar plot (bin width=2.5 deg; see for example, Fig. 2c–f,i,j). Each bin in the histogram was represented as a vector normalized by the total number of boutons formed in all the superficial layer clusters (Fig. 2m,n). The tuning was quantified by taking the sum of all these vectors, normalizing it and representing it as a single line on the same plot (see bold vectors in Fig. 2m,n). Supplementary Figure 5 shows the sum-vectors for each of the 33 neurons. The length of the sum-vector was then considered as a cluster’s tuning. A length of 0 indicates no tuning, that is, the cluster has no orientation bias; 1 represents a cluster tuned to only a single bin of orientation. Figure 2m,n shows that the dendrites and distal clusters of these two neurons have very similar tuning, whereas their local cluster is more broadly tuned (as is evident from the histograms). Distal clusters were more sharply tuned than local clusters, even when the distal cluster lay in very different orientation domains compared with its dendrites (see Fig. 2n). Across the 33 neurons the tuning spanned virtually the entire range between 0 and 1. The normalized mean orientation tuning for the local elipse was 0.6±0.2 (s.d.) and for the distal ellipses it was 0.8±0.2 (s.d.). The sharper tuning of the distal clusters arose because in all but two cases, the local cluster contained the most boutons and spread them over more of the orientation map.

### Cluster rank and SI of all neurons

**Figure 3:**
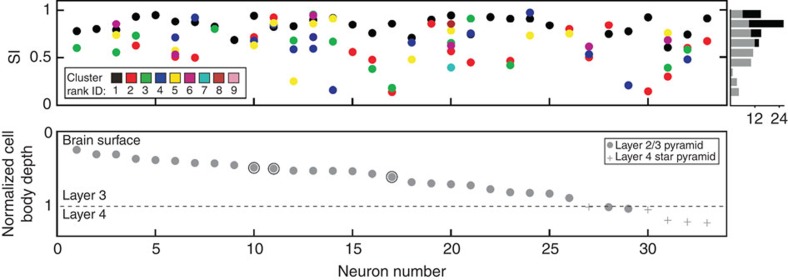
Similarity Index (SI) values for individual clusters of each neuron sorted by normalized depth of soma. (Top) neurons (*x* axis) can have clusters (colour coded by rank) with different SIs (*y* axis). The histogram on the right summarizes the SIs across clusters of all neurons (grey=distal, black=local). Note the large variance within and across neurons. (Bottom) normalized depth from the surface (*y* axis) for each of the 33 neurons: 28 superficial layer pyramids (grey dots) and 5 star pyramids (grey pluses) of layer 4. Circled dots indicate neurons displayed in Fig. 1 (ID 11), Fig. 2 (ID 11/17) and Fig. 5 (ID 10). Note that the distal clusters of neuron 11 have high SIs, whereas those of neuron 17 are very low, and those of neuron 12 and 14 have both high- and low-SI values.

Clusters were ranked according to the number of boutons they contained. Figure 3 shows the SIs of the different rank orders (colour coded, upper plot), plotted according to the normalized depth of the soma in the cortex (grey circles in lower plot). Local clusters (that is, mostly rank 1) tended to have higher SIs than distal clusters (upper plot and histogram). The mean SI for local clusters was 0.85±0.08 (s.d.) (range=0.61–0.96). The mean SI for the distal clusters (that is, predominantly rank 2 and above) was 0.62±0.21 (s.d.) (range=0.13–0.96). Whether the individual bouton clusters were used, or their fitted ellipses, had no influence on the SI (Wilcoxon rank sum, *P*=0.552 (local), *P*=0.904 (distal), see also Fig. 1d,e).

### Size of the cluster and distance from the soma

**Figure 4:**
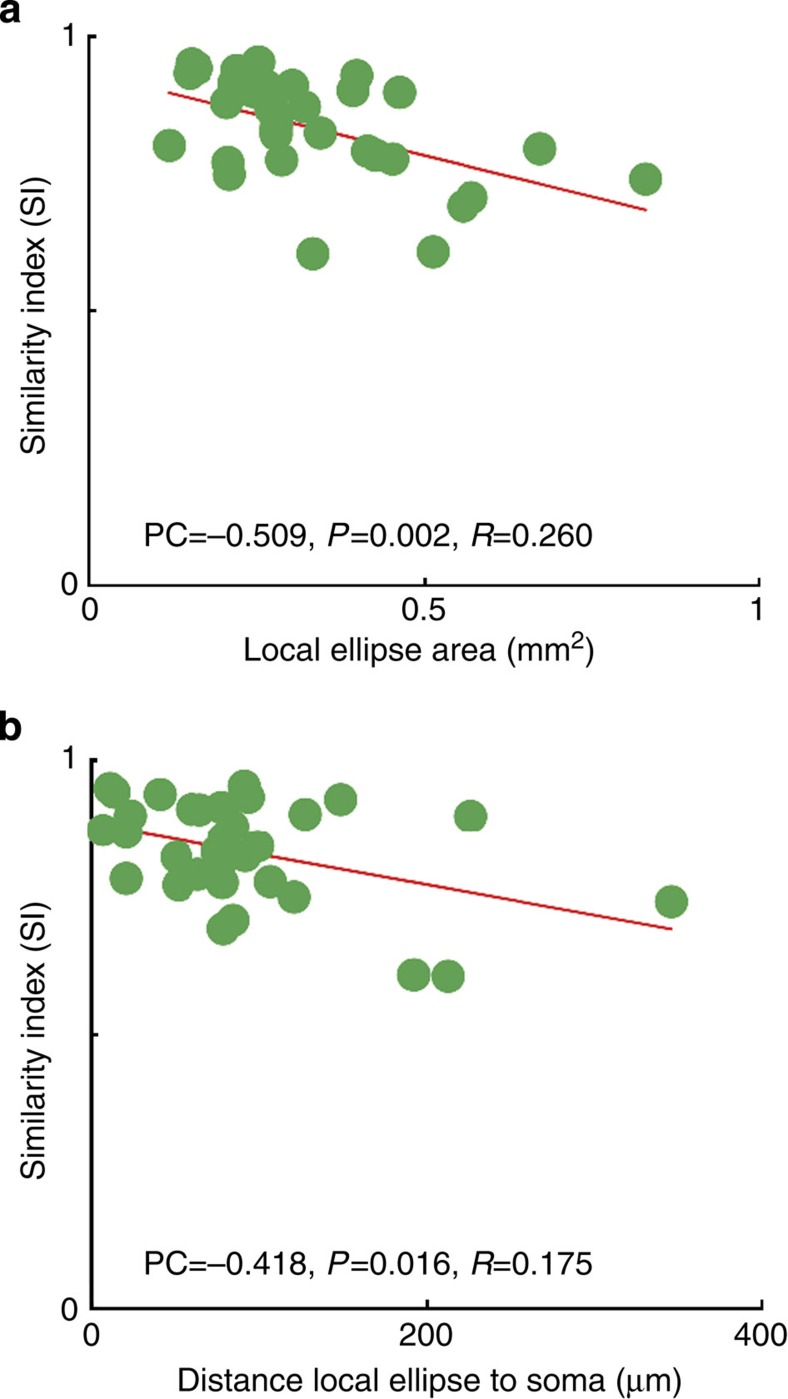
Comparison between the local ellipse’s SI values, their areas and the distance of their centre to the soma. (**a**) The area of each local ellipse correlated negatively with the ellipse’s SI value (Pearson correlation=−0.509, *P* value=0.002 and *R*^2^=0.260, *N*=33), indicating that the bigger a local cluster is the lower is its SI value. (**b**) The SI of the local ellipse’s SI correlated negatively with its distance to the soma (Pearson correlation=−0.418, *P* value=0.016 and *R*^2^=0.175). Hence, the further away the centre of the local ellipse is from its soma the lower is its SI value.

Local clusters formed principally in regions of the orientation map that were most similar to those occupied by the dendritic tree (Fig. 3, black dots and histogram). However, local clusters varied greatly in size and the larger clusters tend to spread over more orientation domains than the smaller clusters (Fig. 4a, see all 33 neuron’s ellipses in Supplementary Figs 6 and 7). Thus the SI was highest for the smallest clusters and lowest for the largest clusters. The local cluster tended to be asymmetrically distributed around the soma position, so we also examined how the the SI (Fig. 4b) related to the eccentricity of the centre of the cluster (determined from the fitted ellipse) from the soma position. There was slight tendency for clusters more centred on the soma to have higher SIs than those whose centres were located more eccentrically (Fig. 4b).

### Pinwheels

**Figure 5:**
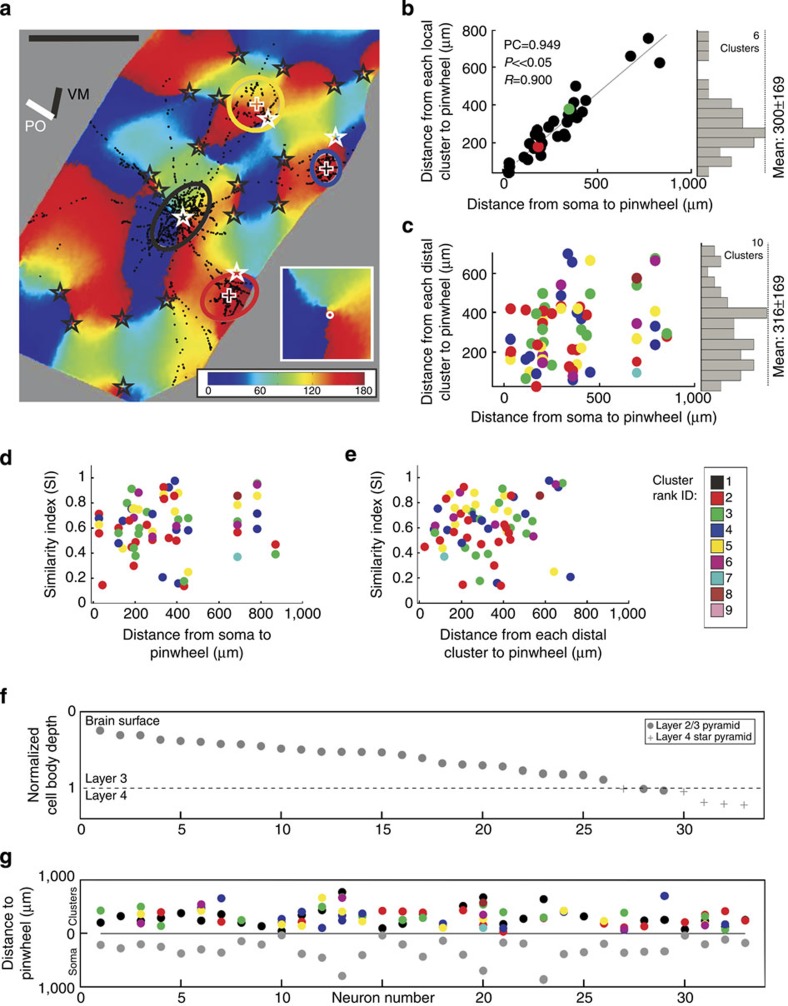
Distance effects of pinwheels. (**a**) Orientation map together with a neuron’s soma located on a pinwheel (Neuron ID=10). Synaptic boutons (black dots) with pinwheel centres (black stars) and cluster ellipses. The white stars mark each ellipse’s nearest pinwheel. The vertical meridian representation (VM) and the neuron’s preferred orientation (PO) are indicated as bars on the top-left. The inset shows a higher magnification of the pinwheel and the location of the soma (white dot). The soma is located on a pixel of the map representing 159 degrees (dark red), similar to the 138 degrees plotted for its preferred orientation. Scale bar, 1 mm. (**b**) Scatterplot of the distance between a neuron’s soma and its closest pinwheel (*x* axis) versus the distance between the local ellipse’s centre and its closest pinwheel (*y* axis). As expected, a significant correlation was observed (Pearson correlation=0.949, *P* value≪0.05 and *R*^2^=0.900, *N*=33). On the right is the histogram of the distances between all neurons local ellipse’s centres to their next pinwheel (mean of 300±169 (s.d.)). (**c**) Scatterplot of the distance between a neuron’s soma and its closest pinwheel (*x* axis) versus the distance between each distal ellipse’s centre and its closest pinwheel (*y* axis). No correlation was observed, indicating that the distance of a distal ellipse to its closest pinwheel is independent of the proximity of the parent soma to a pinwheel. On the right is the histogram of the distances between the distal ellipses centre to its next pinwheel (mean of 316±169 (s.d.), *N*=66). (**d**,**e**) The two scatterplots show the SI value of the distal ellipses (*y* axis) versus the distance between a neuron’s soma and its closest pinwheel (*x* axis, **d**) and the distance between the distal ellipse and its closest pinwheel (*x* axis, **e**). No correlation was observed; indicating that the SI of a distal ellipse is independent of the soma’s or cluster’s proximity to a pinwheel. (**f**) Neurons ranked (*x* axis) according to normalized cell body depth from the surface (*y* axis): 28 superficial pyramids (grey dots) and 5-layer 4-star pyramids (grey pluses). Brain surface and layers as indicated. (**g**) The distance of each ellipse to its closest pinwheel (*y* axis) is plotted against the normalized neuron’s cell body depth (*x* axis). Each coloured dot represents one cluster of a certain rank (see colour table) with its individual distance. The local cluster has mostly the rank 1 (black, see also **b**). Plotted below are the distances of somata to their nearest pinwheel (grey dots). Neuron 10 is shown in **a**. (Neuron ID: 10, cat_2806_RH_neuron_06. Receptive field properties: simple, ocular dominance 5, size 1.3 × 1°, location 2.5°/0° from areal centralis, preferred orientation 138°±21°, direction preference).

One distinctive feature of the orientation map is the presence of pinwheels (see Fig. 5a inset), or ‘singularities’, where all orientations are mapped around a point^11^,^12^. Between the pinwheel centres (stars in Fig. 5a), which are distributed at relatively low density (2.1±2.6 (s.d.) per mm^2^ in our data), are iso-oriented zones. We examined whether the proximity of the neuron to a pinwheel centre influenced the morphology and selectivity of connections. Qualitatively there was no morphological difference between neurons located at or near a pinwheel (for example, Fig. 5a, see all 33 neurons in Supplementary Figs 3 and 4) and those lying in the middle of iso-orientation zones. To compare the 2D map with 3D neurons, the cluster boutons projected onto 2D were fitted with ellipses (Fig. 5a). Self-evidently, the local cluster of a pinwheel neuron was located in the vicinity of a pinwheel centre, but what of the distal clusters? Did they also locate themselves in the vicinity of pinwheel centres? For the example neuron of Fig. 5a, the ellipse representation shows clearly that the local cluster at the pinwheel centre covered all orientations equally, whereas the distal clusters did not. The scatterplot of all distal clusters across all neurons shows that the pinwheel centres did not influence the placement of the distal clusters (Fig. 5c) and did not influence the SI values compared with the proximity of the soma (Fig. 5d) or the distal ellipse to the pinwheel (Fig. 5e). We also found no influence on the distribution of clusters of the cortical layer, or distance of the parent soma from the pinwheel (grey circles, Fig. 5f,g).

**Figure 6:**
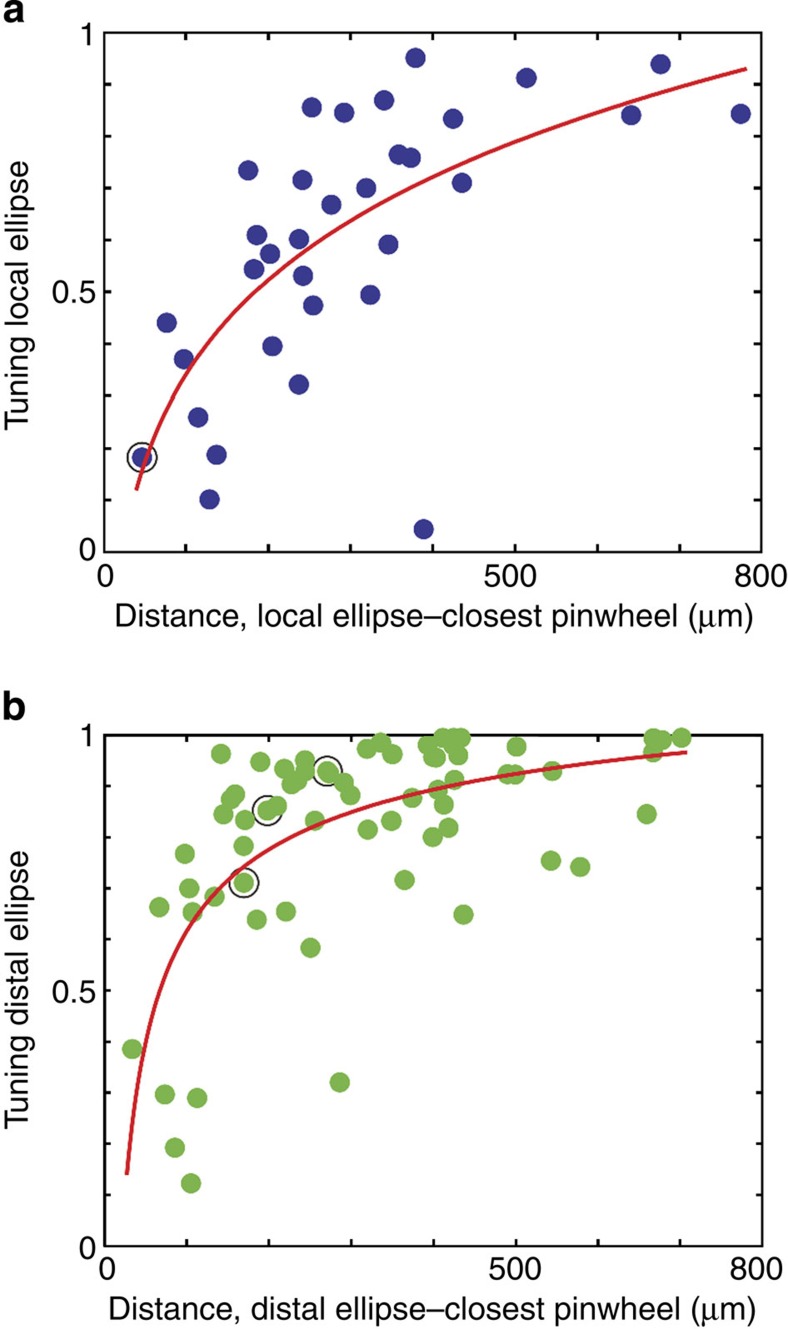
Comparison between the tuning of an ellipse against the proximity to the next pinwheel. (**a**) For each individual neuron the distance between the local ellipse and its closest pinwheel (*x* axis) was plotted against the tuning of the local ellipse (*y* axis). The tuning was calculated as explained in Fig. 2 (see Methods). The points were fitted by an exponential function of the form a*xb+c (SSE: 1.1289, *R*^2^: 0.4534, *N*=33). The data indicate that tuning of the local ellipse decreases with proximity of a neuron’s soma to a pinwheel. (**b**) Same as in **a** for only distal ellipses (SSE: 1.6000, *R*^2^: 0.4150, *N*=66). As with local ellipses, their tuning decreases with proximity to a pinwheel. Encircled dots in **a** and **b** are from neuron No. 10, shown in Fig. 5a.

As expected, there was a relation between the tuning of the ellipse and the distance of the cluster from a pinwheel centre (Fig. 6). The closer the cluster was to the pinwheel centre, the broader was its orientation tuning, that is, the cluster showed no evidence of increasing its specificity for any particular orientation (including its parent cell’s) in the region of the pinwheel, but distributed itself across all orientation domains of the pinwheel. The tuning of the distal clusters was similarly positively correlated with their proximity to pinwheels (Fig. 6b). These observations suggest that the extent and placing of local and distal clusters are not influenced by the presence of pinwheels.

### Retinotopy

**Figure 7:**
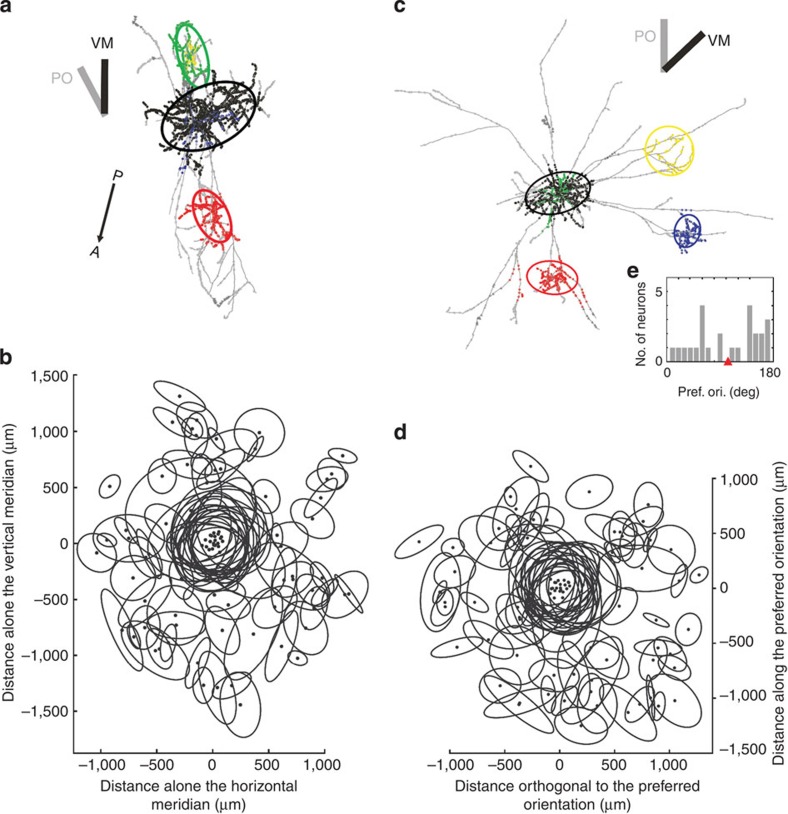
Superposition of superficial layer cluster ellipses across the 25 neurons whose RFs were plotted. (**a**,**c**) The neurons are shown in their top-view together with the angle of the vertical meridian representation (VM, black bar) and the neuron’s preferred orientation (PO, grey bar). Posterior–Anterior (P–A) arrow indicated stereotaxic midline (the crucial axis to align and overlay all neurons). Boutons belonging to different clusters (and their fitted ellipses) are colour coded by different colours (black, red, green, blue, yellow and so on in order of increasing cluster rank). Boutons outside clusters are marked in grey. (See all 33 neurons individually in Supplementary Fig. 8). (**b**,**d**) Superposition of all ellipses of superficial layer clusters. Each neuron was rotated and aligned to a common coordinate system of the vertical meridian, **b**, or its preferred orientation, **d**. Both graphs highlight that clusters are irrespectively formed to the two functional axes (vertical meridian and neurons preferred orientations). (**e**) Distribution of preferred orientations for the corresponding 25 neurons (see more details in Supplementary Table 1). The red triangle marks the mean. (Neuron IDs: **a** (17), **c** (10)).

Many studies have looked for anisotropies in the overall tangential alignment of the distal clusters. The clearest example was found in area 17 of the tree shrew where bulk-labelled axons elongated along the retinotopic projection of the preferred orientation of the source neurons, and clusters were found to connect preferentially to distal ‘like’ orientations^6^,^13^. To examine these relations at single-neuron resolution, we placed all the cell somata for which we had RFs (*n*=25) on the same point and plotted all their superficial layer clusters with reference to the projection of vertical meridian (VM, see Methods). The example neuron in Fig. 7a shows that while its local cluster was oriented obliquely to the representation of VM, the overall alignment of its clusters was along the VM representation. However, the scatterplot of the ellipses of all 25 neurons showed that all angles were represented with no bias to any particular axis (Fig. 7b, see all 33 neurons individually in Supplementary Fig. 8), independent of the rank order of the cluster. Thus clusters in cat V1 do not have any special alignment with the cardinal visual axes.

A similar analysis was made taking the orientation preference of the neuron as the common reference, again placing all somata on the same point. The example neuron shown in Fig. 7c had an orientation tuning indicated by the grey bar, and two of its three distal clusters were not aligned along this axis. The orientation preferences of the RFs (plotted through the dominant eye) of the 25 neurons were distributed around the clock (Fig. 7e; Supplementary Table 1). The scatterplot of Fig. 7d, however, shows that the clusters do not preferentially align along the axis of the preferred orientation of the parent neuron. The cluster rank also showed no correlation with the alignment of the preferred orientation (see Supplementary Fig. 8). The same was true when considering only boutons, instead of the ellipses (see Supplementary Fig. 9). Thus clusters in cat V1 do not have any special alignment with the retinotopic representation of neurons’ preferred orientations.

### Bootstrap

The above analyses showed that, unlike the tree shrew^6^, the distribution of the clusters analysed in cat area 17 is not constrained by the retinotopic map or by the orientation preferences of the neurons. We also found that the distal clusters of individual neurons showed a wide range of SI values. This raises the more general question of whether clusters just distribute themselves randomly, or according to some pattern. We tested the measured SI against a distribution of many possible random SIs (up to 20,000).

**Figure 8:**
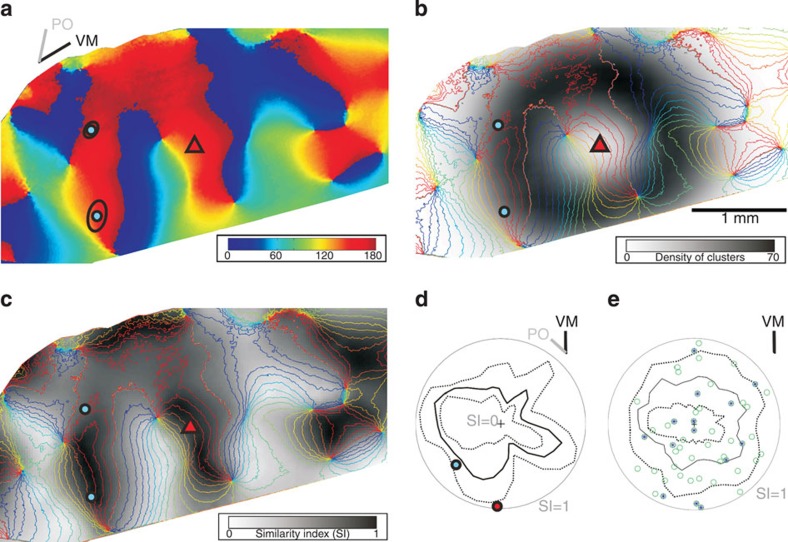
Bootstrapping to test whether bouton clusters were positioned randomly. (**a**) Orientation map with cell soma (red triangle) and two distal cluster’s ellipses (black curves) with their centre (cyan dots). Colour code for orientation angle indicated in strip. Bars (top-left) indicate preferred orientation (PO) of the neuron and the angle of vertical meridian (VM) representation. (**b**) Smoothed density distribution for 20,000 bootstrapped cluster centres (see Methods) overlaid by a contour plot (10-deg intervals) of the orientation map shown in **a**. Greyscale values denote the cluster density. (**c**) 2D Similarity Index (SI) distribution after 20,000 bootstrapped clusters. The SI values are greyscale coded where black is an SI of 1 and white an SI of 0. (**d**) Clusters whose centres lay within the local ellipse or which fell outside the map were ignored. The remaining clusters were used to test whether actual clusters were randomly placed. Confidence intervals for each circular sector (bin width 10 degrees) centred at the cell soma were calculated for all the SIs in **c**. Radial distance from centre (black cross) gives magnitude of SI value (grey circumference indicates SI=1), and the circular angle denotes the sector’s orientation in space. Dashed lines denote the 5% and 95% percentile confidence intervals. The median is indicated by a solid black line. If an SI value of an actual measured cluster was outside the confidence interval boundaries (for example, red circle) the cluster placement was considered as significantly different from what would be expected from a random placement of clusters. (**e**) Superposition of all superficial layer distal clusters of the 25 neurons individually aligned to their vertical meridian. Clusters lying outside their confidence interval are marked with stars. (Neuron ID: 24, cat_0707_RH_neuron_01. Simple 2 RF, ocular dominance 3, size 1.4 × 1.4°, location 0.7°/−3.3° from areal centralis, preferred orientation 138°±17°, direction selective 228°, 75 MΩ). (See the same procedure exemplified for another neuron in Supplementary Fig. 10).

The orientation map and the two distal cluster ellipses are shown for an example neuron (ID 24) in Fig. 8a. The density of 20,000 bootstrapped cluster centres is plotted in a smoothed greyscale map overlaid with the contours of the orientation map (Fig. 8b). The smoothed distribution of SIs from all bootstrapped ellipses are shown along with the contours of the orientation map (Fig. 8c). Then, bootstrapped clusters with their centres within the elipse of the local cluster, or which lay outside the orientation map, were ignored for further comparison.

The SI for each of the clusters of the actual neuron was compared with the distribution of SIs of all the bootstrapped elipses within one radial sector (see polar plot in Fig. 8d; the radial dimension represents the SI and the grey outer circle indicates the SI of 1). The circular angle of the polar plot indicates the circular direction of the centre of the ellipses (bin width 10 deg). Indicated in colours are the centres of the actual fitted ellipses, the median (black line) and the 5%/95% percentiles (dotted lines) of the bootstrapped SIs. The percentiles were determined for each sector from all bootstrapped values in that sector on the basis of the density (Fig. 8b) and the similarity (see Fig. 8c). In each radial direction the contours of the polar plot of the SIs follow the dominant values of the orientation map (Fig. 8a) in that sector. For this particular neuron, one of the two distal clusters had an SI that was significantly greater than expected by chance (marked by a red dot, Fig. 8d). Supplementary Figure 10 exemplifies the bootstrap procedure for another neuron. The bootstrap procedure was repeated for each of the 25 neurons, aligned with the retinotopic map (Fig. 8e). Out of 51 clusters, 28% (14 clusters) were located in positions that were significantly different from random (that is, lay outside the percentiles), independent of the cluster’s SI value, its location from soma, its relation to the VM (Fig. 8e) or to the preferred orientation (see Supplementary Fig. 10l). This indicates that a significantly high fraction of 28% of all superficial layer distal clusters were located in positions that were unlikely to occur by chance.

## Discussion

The lateral clusters of boutons formed by superficial layer pyramidal cells are a ubiquitous, if enigmatic, feature of the neocortex of higher mammals^3^,^14^. With rare exceptions^15^, however, previous studies have analysed the ‘average’ projection pattern resulting from bulk labelling of many neurons. This method labels indiscriminately smooth and spiny neurons and does so both retrogradely and anterogradely and thus may not give an accurate low-resolution picture even of the average connectivity of the lateral pyramidal cell network. Nevertheless, in V1, these data, arising from a relatively small number of experiments, have been interpreted as strong evidence for a like-to-like rule that links iso-orientation patches of the orientation map^5^,^6^,^15^,^16^,^17^,^18^,^19^,^20^.

Neurons in the primary visual cortex are tuned to multiple visual features (for example, orientation, length, depth, contrast, direction preference, spatial and temporal frequency), some properties such as orientation tuning and ocular dominance are organized in regular maps^21^, other properties such as depth and temporal frequency are not. The orientation map is by far the most robust of all functional maps in the cat and thus orientation has been the parameter of choice in studies similar to ours.

One key issue for our interpretation of structure–function correlations is whether the intrinsic imaging, derived as it is from blood flow, really does map faithfully the orientation selectivity of the clusters of neurons? Fortunately, the close correlation of orientation tuning determined by single-unit electrophysiology and the orientation map derived from the intrinsic signal has now been validated in many studies^22^,^23^,^24^, including ours.

Calcium imaging with two-photon microscopy can give the precise locations of single physiologically characterized cells, and thus would seem preferred to the lower-resolution techniques of voltage-sensitive dye (VSD) imaging or intrinsic signal imaging we used, however, the field of view offered by the two-photon microscope is typically an order of magnitude smaller than was necessary for our study. Hence intrinsic signal imaging, which offers a large field of view, was essential. For similar reasons of scale, the method used effectively in mouse visual cortex to identify synaptic connections between neighbouring neurons, that is, calcium imaging *in vivo* followed by paired recording *in vitro*^25^, or serial section electron microscopy^26^,^27^, unfortunately cannot be applied in cat. Slicing the cortex for *in vitro* recording or serial-section electron microscopy severs far more of the complex lateral axon arbors of the pyramidal cells of higher mammals compared with their rodent equivalents. Being able to locate the single cells in relation to other fiducial points, and using a processing protocol that reduced shrinkage to a minimum, greatly ameliorated the problems of registering the intrinsic signal images obtained *in vivo* with the reconstructed histology. In addition, by recording on top of the gyrus and windowing the imaged area, we reduced the distortion due to gyral curvature to a few percent. Hence, we can be confident that the ‘SI’ gives an accurate quantitative measure of the orientation domains sampled by dendrites and bouton clusters.

Given previous studies, the high variance in the SI, both for the clusters of single cells and across the samples, was unexpected. It indicated that the distal domains of the axon spanned virtually the entire range of possible domains of the orientation map (SI values range 0.13–0.96), with only a weak average bias to like-to-like coupling. Moreover, our analyses showed that the tuning of the neuron’s dendritic tree, the tuning of the distal bouton clusters and the neuron’s SI, were all independent of one another. Our bootstrap procedure indicated that a significant high fraction of 28% of all distal clusters were located in positions that were unlikely to occur by chance. Thus the placement of clusters seems to be a result of a deliberate wiring strategy, not simply noise. The picture that emerges therefore, is rather different from that of previous studies, where the neurons were thought to link iso-orientation zones, albeit with very poor precision since only about 60% of the clusters lay in or near orientation domains that matched that of the cell body^5^,^6^,^15^,^16^,^17^,^19^,^20^. (Unsurprisingly, if we average the distribution of distal clusters across all 33 neurons, we also find a similarly poor precision—an average SI of 0.62.) The structural observations of cat V1 are consistent with the physiological observations of Chavane *et al.*^28^ who used a combination of intracellular recording and VSD imaging in cat V1 to show that the lateral spread of the VSD does not respect orientation domains, and that subthreshold responses arise from all orientations. Closely related findings have recently been reported by Huang *et al.*^29^ for V1 in the tree shrew.

In the tree shrew^6^ and new world monkey^7^ the overall patterning of clusters after bulk extracellular labelling was ellipsoid, with the long axis aligned along the axis of orientation preference of the neurons. This pattern was not reflected in our data, where the multiple distal patches formed by single axons were not aligned with the iso-orientation axis or with the representation of the VM. Instead a single axon could branch to innervate sectors of the retinotopic map that were quite different, even orthogonal, to the neuron’s iso-orientation axis.

It has been proved difficult to demonstrate a direct causal link between the lateral clusters and RF structure or perceptual phenomena. Chisum *et al.*^30^ used optical recordings of the intrinsic signal in V1 of the tree shrew to explore the effects of small Gabor patches, presented singly, or in multiples presented either co-linearly or non-co-linearly. Perhaps disappointingly, the experiment with multiple patches also showed no evidence that the hot spots generated by a single patch (the ‘feedforward imprint’) were ‘joined up’ by the lateral connections. If anything, the activation generated by any one of the multiple co-linear patches was more restricted than for the single Gabor, suggesting perhaps some form of competition between the neurons representing different elements of the stimulus.

In V1, the lateral connections have been discussed primarily in terms of Gestalt perceptual phenomena^31^ and other processes like feature binding and scene segmentation through promotion of synchronized oscillatory activity^30^,^32^ and representation of focal orientation discontinuities such as junctions or corners^33^. It is not credible that all these mechanisms derive from simple like-to-like connections between orientation domains. Thus, instead of treating the lateral connections as a single homogenous network, the real clue to its structure and function may lie in its heterogeneity of connections. The lateral network we have described enables each neuron to sample from a variety of functional domains through specific connections from their presynaptic partner, each of which codes for multiple features, and this provides the required substrate for context-dependent processing of natural scenes^34^. This process requires the integration of information from multiple orientations^28^,^35^, the detection of boundaries defined by feature discontinuities, not contrast^36^.

One issue we have not been able to address here is the identity of the postsynaptic elements. The ultrastructural results of several investigations, however, indicate that the superficial layer pyramidal cells in cat and monkey connect mainly with the dendritic spines of other pyramidal cells^37^,^38^,^39^,^40^, with a minority of synapses formed with smooth GABAergic neurons. Fortunately, our interpretations of the functions of the local and lateral connections do not depend on knowing the precise identity of the target neurons or the precise location of the synapses on the dendritic tree. Our major new observation is that not just a local group, but individual neurons can connect to a wide variety of orientation domains (and, we might infer, a similar heterogeneity of other spatio-temporal properties) and it is this heterogeneity that needs to be explained.

Heterogeneity in the lateral connections may be essential for achieving sparse coding through decorrelation in a columnar cortex. In this context, we recently found very low signal and noise correlations in spike firing between adjacent neurons in cat V1 across a wide range of artificial and natural stimuli^41^. It also explains, for example, how simple juxtapositions of orientations could be at the heart of illusions such as the Hering Illusion, where straight parallel lines appear to bend when intersected with oblique lines. The significance that individual neurons sample across a wide array of specific inputs is that it provides the means of contextualizing their responses and a means of feature-independent normalization of the whole network, as occurs for, for example, contrast adaptation^42^,^43^. This means that while a given neuron shares the properties of its neighbours for some mapped properties, such as orientation selectivity, the expression of those mapped properties are modulated by a unique combination of inputs that are active for each particular context, so permitting combinations of contextual cues to influence appropriately the processing of objects in natural scenes. Given the ubiquity of such a network across the neocortex of higher mammals, the cortical daisy may provide a general framework for context-dependent processing by the local cortical circuit.

## Methods

### Species and selection of neurons

The neurons examined in this study were obtained from 1–3-year-old adult cats of both sexes that had been prepared for *in vivo* intracellular recording under general anaesthesia^8^,^44^. All experiments were carried out with authorization under licences granted to KACM by the Kantonales Veterinaeramt of Zurich. For 15 hemispheres (of 13 cats), the orientation maps were acquired by optical imaging of the intrinsic signal (see for example, Fig. 1). From 231 recording sites, 50 of the 153 neurons impaled had a well-filled axon. After excluding neurons having inferior corresponding optical imaging maps, we arrived at a final data set of 33 neurons, 25 of which had hand-plotted RFs (see Supplementary Table 1). Pyramids and star pyramids were pooled unless a significant difference was encountered.

### Surgery

The cats were prepared for surgery after the administration of subcutaneous premedication of xylazine (Rompun, Beyelar, 0.5 mg kg^−1^) and (Narketan 10, Vetoquinol AG, CH, 10 mg kg^−1^). Initial surgery was performed under additional gas anaesthesia using 1–2% halothane (Arovet AG, CH) in oxygen/nitrous oxide (50%/50%). After induction of general anaesthesia, the femoral vein was cannulated and alphaxalone/alphadalone (Saffan, Glaxo) delivered to establish complete anaesthesia during the remainder of the experiment. The femoral artery was cannulated to measure blood pressure. After a tracheotomy the cat was moved to a stereotaxic apparatus, where it was respirated artificially with a mixture of oxygen/nitrous oxide (30%/70%). Halothane (0.5–1.5%) supplemented the intravenous anaesthesia when needed. The respiratory pump volume was adjusted to constant 4.5% end-tidal CO_2_. Lidocaine gel (4%) was applied to all pressure points. Electroencephalogram, electrocardiogram, heart rate, blood pressure, end-tidal CO_2_ and rectal temperature were monitored continuously during the entire experiment. A thermistor-controlled heating blanket maintained the cat’s rectal temperature at 37 °C. Topical antibiotics (Voltamicin, Novartis) and Atropine 1% (Novartis) (to paralyse in accommodation) were applied to the eyes before they were covered with gas-permeable contact lenses. To retract the nictitating membrane phenylephrine 5% (Blache) was used. A craniotomy was performed over area 17 from Horsley–Clark coordinates AP −1 to −9 and LM from the midline to 5 mm lateral. A plastic chamber was mounted over the craniotomy and fixed to the bone with dental cement. After the craniotomy, the cats received an intravenous injection of the muscle relaxant gallamine triethiodide (40 mg induction dose) (Sigma Aldrich, CH) followed by a continuous infusion of gallamine triethiodide (13 mg kg^−1^ h^−1^) and (+)-tubocurarine chloride hydrate (1 mg kg^−1^ h^−1^) (Sigma). The eyes were refracted and lenses added to focus the eyes on a plotting board.

### Recording

Glass micropipettes were used for intracellular recording. A small durotomy was made for each penetration, which was noted on a drawing of the pattern of blood vessels. RFs were handplotted during extracellular recording and replotted during intracellular recording to be sure it was the same neuron (it invariably did). Then horseradish peroxidase was injected by iontophoresis^44^. Across all 231 recording sites, one neuron had an average RF size of 1.7 × 1.9 deg and an average eccentricity of 3.1 deg, which corresponds to a cortical magnification factor at that eccentricity between 0.8 mm per deg and 1.9 mm per deg^45^.

### Optical imaging

To capture the activity of a whole region of cortex, one can combine presentations of full-field moving oriented gratings with the method of optical imaging of intrinsic signals. These signals arise from changes in cortical reflectance linked to metabolic changes, such as increased blood flow, oxy/deoxy haemaglobin ratio and light scattering resulting from local neural activity due to the oriented gratings. This way one can determine the orientation preference of a whole population of neurons and can thus generate a functional map for the whole imaged region of cortex. To acquire such optical imaging data, we used the standard optical imaging protocol^21^,^46^. Our camera (CS8310BC, Teli, Japan) recorded from 600 μm depth and data acquisition was done with the Imager 3001 VSD+/VDAQ setup (Optical Imaging Inc.). Visual stimuli consisted of square wave gratings of eight different orientations (0°, 22.5°, 45°, 67.5°, 90°, 112.5°, 135° and 157.5°) with 100% contrast, spatial frequency of 1 cycle·per degree and temporal frequency of 1 degree s^−1^. During inter-stimulus intervals, the next stimulus, randomly selected, was presented as stationary. After excluding blood vessels, single-orientation maps were calculated with either the ‘cocktail blank’ method or by dividing maps encompassing orthogonal responses^47^. Next, the single-orientation maps were vectorially summed on a pixel-by-pixel basis and the resulting angle was then rounded to the closest orientation of the presented gratings^12^. This generated one colour-coded image representing all orientations at once, termed the ‘orientation map’. All calculations were performed in Matlab (MathWorks Inc.). See a review of the method of optical imaging by Zepeda *et al.*^48^

### Alignment of brain sections with the orientation map

Before the perfusion, up to six 2-mm deep reference penetrations were made with empty glass pipettes in the stereotaxic coordinate frame and their positions were noted on the blood-vessel pattern. Then the shaft was cut across a few mm above the brain surface and the rest remained in place for perfusion^49^. After perfusion, the glass pipettes were withdrawn leaving equally sized tracks (20–50 μm) in the brain tissue that could later on be identified in histological sections (Supplementary Fig. 11). These equally sized tracks were used to align the 3D neurons with the orientation maps. See all 33 neurons aligned with their corresponding orientation map in Supplementary Figs 6 and 7.

### Fixation and histology

The block of tissue containing the intracellularly filled neurons was serially sectioned (80 μm) in the horizontal plane, processed to reveal horseradish peroxidase and finally osmicated and embedded in resin to reduce differential shrinkage^8^. This allowed examinations at both the light and the electron microscopic levels.

### Reconstructions

Individual neurons were reconstructed in 3D using a microscope ( × 100, Olympus BX-51) combined with a motorized stage (MicroBrightField Inc., USA) and the aid of the Neurolucida software (Version 8.0, MicroBrightField Inc.). See all 33 neurons in their top-view in Supplementary Figs 3 and 4. The mentioned reference penetrations and various fiducial marks (for example, blood vessels) were used to align the serial sections. While reconstructing the axon (~100 h per neuron) each bouton encountered was tagged with a marker. Further, all layer boundaries were digitized. In addition the anterio-posterior axis, that is, midline, was tagged with two markers called ‘Anterior’ and ‘Posterior’ that were crucial to later on align the whole 3D reconstruction to the VM. Finally, the brain surface, cell body, reference penetration, layer boundaries and the markers Anterior and Posterior were saved together with the dendrite, the axon and the boutons as an XML data file for further analysis.

The software Neurolucida has limited tools for data analyses. Therefore a framework was created in-house called ‘Nereda’ by Dr S. Roth to analyse the XML data (see Supplementary Material).

### Selection of best fill neurons

Out of 231 recording sites, we attempted to label intracellularly 153 individual neurons. Of these 153 impaled neurons, 65 had a well-filled dendritic arbor and axonal trunk thus we reconstructed them in 3D. Although intracellular fills are thought to be complete, we were concerned to estimate their ‘completeness’ by noting the nature of the ends of every single axonal branch. Each ending of an axonal branch was classified as either a ‘normal’ ending, or an ‘incomplete’ ending. The normal ending was characterized by a bouton with no subsequent axonal processes, or when the end of the segment formed an abrupt high-contrast ending without fading out. In all other cases the ending was classified as incomplete. Therefore each axonal segment that had no subsequent axonal processes, or terminal segment, had a distinct ending-type: either complete or incomplete. After finishing the reconstruction of 65 neurons, we removed 15 on the basis of their number of incomplete terminals.

### Creating volumes out of the layer boundaries

The 2D layer boundaries resembled contour lines of a hill in a topographic map, but to quantify any particular layer affiliation in 3D, these lines had to be converted into volumes. The 2D layer boundaries were first converted into surfaces by rolling a virtual ball of a certain size over the contour lines thus creating a triangulated surface. This triangulated surface created a subjacent volume that was further discretized in voxels (voxel size 20 μm). Now data points could be affiliated in 3D to a certain voxel, which belonged to a certain volume under a specific layer boundary. This allowed a very precise exclusion of deep-layer processes in 3D and a pure focus on the superficial layers.

### Mean-shift bouton clustering

To achieve an objective means of defining the 3D bouton cluster, Binzegger *et al.*^10^ developed a mean-shift cluster-algorithm, which was applied to each of our investigated neurons. Clusters were ranked according to the number of boutons they contained. Typically, the local cluster was rank 1 and the succeessively smaller distal clusters were ranked successively higher. See Supplementary Fig. 1 how the algorithm identifies bouton clusters and see all 33 neurons in Supplementary Figs 3 and 4. It is important to mention that the key feature of the ‘Daisy’ is that there are clustered regions and the sparse regions between, that is, the linear regions containing axons that extend laterally to form clusters. Thus it is crucial to exclude such linear regions from any cluster analysis as they will obivously falsify the results and conclusions.

### Parameterization of bouton clusters

For simple parameterization, the identified 3D bouton clusters were fitted by a 3D ellipsoid and thus allowing straightforward 3D analysis (for example, the angle of a bouton cluster versus brain surface). For analysis in 2D (for example, comparisons with the orientation map) the bouton clusters were projected on a single-projection plane and then fitted by a 2D ellipse and thus also parameterized. See two examples in Fig. 7a,c and all 33 neurons in Supplementary Fig. 8. We termed a 3D ellipsoid simply as ‘ellipsoid’ and a 2D ellipse simply as ‘ellipse’, so not to be confused.

### The projection plane

To take the typical gyral curvature of A17 into account^45^, a ‘projection plane’ was created for each individual neuron. This projection plane was orthogonal to the vertical axis centred on the soma. Thus the projection of an individual neuron onto their projection plane can be thought as an azimuthal projection, hence making pure 2D analyses meaningful (see all 33 neurons in their azimuthal projection in Supplementary Figs 3 and 4). Then, to determine any direction bias of an axon, these azimuthal projections were rotated by 14° relative to the midline (determined by the two markers Anterior, Posterior). This allowed the final alignment of a neuron with the VM representation of the average retinotopic map^50^. Obviously the projection plane is not necessarily equal to the plane of focus of the macroscope (compare each neuron’s ellipses in Supplementary Fig. 8 versus Supplementary Figs 6 and 7).

### Combining maps and reconstructions

The reconstructions of each hemisphere (*n*=15) were rotated in 3D until the point of view matched the view of the macroscope. This rotation was defined by (1) the spatial orientation of the stereotactically aligned reference penetration tracks and (2) the measured angle of the macroscope relative to the stereotaxic plane (see Supplementary Fig. 11).

### Processing of maps

During perfusion the brain tissue shrank. Thus the optical maps had to be linearly transformed with the aid of two sets of points: (1) the marked reference penetrations on the blood-vessel pattern and (2) the entry points of the reference penetration tracks in the reconstructed 3D tissue. Then regions were masked where (a) the blood-vessel pattern was out of focus, (b) the quality of the orientation maps was inferior and (c) axonal processes were encountered down the medial bank or the lateral sulcus (see all 33 neurons overlaid on their corresponding orientation map in Supplementary Figs 6 and 7). Dr Dylan Muir calculated for a curvature between 4 and 8 mm a maximum distortion of 7% for domain width and 2% for domain spacing.

### Method of calculating the SI

The mean-shift algorithm allowed us to identify individual bouton clusters objectively. We then developed a new method called CDF to calculate for each individual bouton cluster (the single local cluster and the multiple distal clusters) a ‘SI’. This index indicates the similarity between each neuron’s individual cluster and the neuron’s own dendritic tree in the domains of the orientation map they cover.

### Cumulative Distribution Function (CDF)

The CDF uses the orientation map, the dendritic tree (subdivided in 1-μm-long segments) and the bouton clusters (see Fig. 2). Each individual bouton in a cluster was given the orientation preference of its underlying pixel in the orientation map. The same procedure was applied to all the dendritic segments.

To calculate the SI, a modified Kolmogorov–Smirnov test (KS test) was applied. As in the KS test, two curves were used: one derived from the cumulative orientation values of the segments of the dendritic tree, and one derived from the cumulative orientation values of all the boutons in a given cluster. But then instead of taking the maximal deviation as in the KS test, we calculated the area A between the two curves. Intuitively, a small area between the two curves indicates a high similarity between the two profiles (dendrite versus cluster) meaning the axon innervates the same orientation domains as the dendrite.

The maximal possible area between any two curves is 180 degrees. But because the orientation map values are circular (0 degrees is equal 180 degrees) the maximal possible area is only 90. Thus the formula to calculate a bouton cluster’s SI is: SI=1−(A/90).

If the area A is maximal (for example, 90, no similarity) the SI becomes 0 and vice versa.

This method was applied to both the bouton cluster and its fitted ellipses. In the latter case the distribution of orientation map values was based on all the pixels encircled by the ellipse (see Fig. 2).

### Pinwheels

The orientation map contains appealing sites called ‘pinwheels’^11^,^51^. The coordinates of every such pinwheel centre were extracted and used to calculate the distance from a neuron’s soma or cluster to its closest pinwheel (see Fig. 5).

### Bootstrap analysis

Distal clusters of one individual neuron could express different SIs and one possibility is that they form at random locations with respect to the functional maps. To explore whether that is the case, we developed a bootstrap analysis based on the orientation maps and the morphological features of individual distal bouton clusters. The features of the individual bouton clusters were derived from all our 50 well-filled neurons (incl. those lacking optical maps) and have the following characteristics: (A) the mean distance between the soma and the centre of the distal cluster was 942 μm±332, 2 (s.d.). (B) The fitted ellipsoids were angled at a mean of 30 degrees±18 (s.d.) from the brain surface. This small angle indicates that the shape of distal clusters, seen from the brain surface, can be simplified by projecting the ellipsoid into an ellipse. By doing so, only little of its original dimension is lost. (C) The projected ellipses were defined by the semi-major (211 μm±74 (s.d.)) and semi-minor axis (107 μm±49 (s.d.)). (D) The ellipses were circularly irregularly placed with respect to the soma and did not aggregate along the neurons individual preferred orientation or any other retinotopic axis.

In summary, we can assume that when looking from the brain surface, individual distal cluster can be parameterized simply by considering only the ellipse with its radial dislocation from the soma (A) and the length of its semi-axis (C). Thus parameters A and C allowed us to generate realistic ellipses and place them randomly on the functional map.

For every neuron that had an associated high-quality orientation map (*n*=33), the bootstrap analysis (see Fig. 8) comprised the following: take all 275 measured distal ellipses (parameterized through fact C from all 50 neurons), select randomly one, calculate a new radial dislocation from soma (based on fact A with normal distribution, mean=942 and s.d.=332, see. Fig. 8b), rotate the ellipse individually by a random angle (uniform distribution in [−pi pi]) around its centre and calculate finally its SI. This procedure was repeated 20,000 times for each neuron. Those ellipses having their centre either outside the orientation map or inside the local ellipse were excluded for further analysis.

To test whether our observed individual clusters are predictable or not we binned the bootstrapped data in radial circular sectors (10 degree bin width). We then took from each of these radial sectors all its bootstrapped SI values and created a distribution. This distribution defined the 5 and 95% percentiles and thus enabled us to do the statistical test if a real cluster’s SI is within the expected range or not. If not, it was termed as statistically significant, that is, the real cluster was significantly outside the expected distribution of SI values in that particular radial sector (see Fig. 8d). See the same procedure exemplified for another neuron in Supplementary Fig. 10.

In addition, to sum up across all neurons and to see whether there are any correlations in respect to the meaningful retinotopic axes, all radial sectors and their SI-distributions were independently rotated two times: one time to fit the VM representation (see Fig. 8e) and one time to fit the neuron’s own preferred orientation (see Supplementary Fig. 10l).

### Statistical analysis

Parameters are quoted by their means and s.d. Pearson correlation was used to test for significant linear relationships, and if not stated differently, Wilcoxon sign rank tests were used for all other tests (all tests alpha=5%).

## Author contributions

K.A.C.M., S.R. and E.S.R. contributed equally to ensuring the success of the design, *in vivo* experiments, post-processing and analysis and drafting of the typescript.

## Additional information

**How to cite this article:** Martin, K. A. C. *et al.* Superficial layer pyramidal cells communicate heterogeneously between multiple functional domains of cat primary visual cortex. *Nat. Commun.* 5:5252 doi: 10.1038/ncomms6252 (2014).

## Supplementary Material

Supplementary Information Supplementary Figures 1-11, Supplementary Tables 1-2 and Supplementary Methods
