# Supplementary material for: Superficial layer pyramidal cells communicate heterogeneously between multiple functional domains of cat primary visual cortex
Source: Nat Commun. 2014 Oct 24;5:5252. doi: 10.1038/ncomms6252 (PMC4354012; doi:10.1038/ncomms6252)
Supplement: Supplementary Information — Supplementary Figures 1-11, Supplementary Tables 1-2 and Supplementary Methods [file ncomms6252-s1.pdf]

Superficial layer pyramidal cells communicate heterogeneously between multiple functional domains of cat primary visual cortex.

Kevan AC Martin, Stephan Roth, Elisha S Rusch

Supplementary Material

A. Example of a typical superficial layer pyramidal neuron.

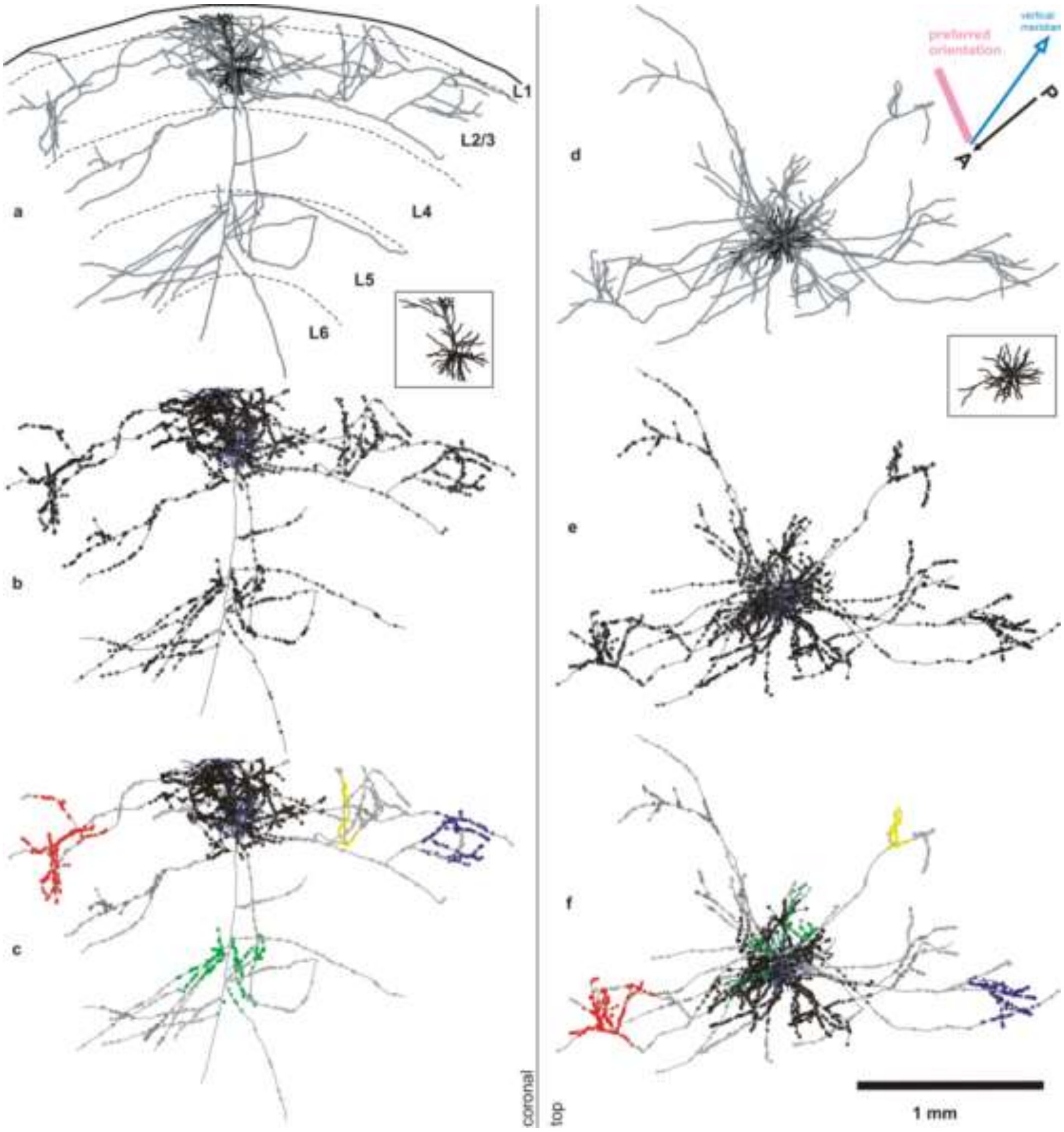

Supplementary Figure 1 Example of a typical superficial layer pyramidal neuron. (**a-c**) shows the side view and (**d-f**) the top view of the reconstructed axonal tree, boutons and dendrite. (**a**) The brain surface and the layer boundaries are depicted with black curves, the ID's of the 6 different lamina are indicated with the abbreviations L1 - L6. The axonal tree (grey) forms extensive lateral connections in the supragranular layers and minor bifurcations within layer 5. The dendritic tree (black) bifurcates locally and forms one apical dendrite towards layer 1 to form a tuft (better visible in the inset containing the single dendrite). (**b**) Axonal boutons are denoted by enlarged black dots forming high and low density regions within layer 2 and 3 and layer 5. These regions of high bouton densities are captured by the use of a mean-shift cluster-algorithm (applied on the 3D data, see Methods). (**c**) This mean-shift cluster-algorithm extracted five discriminated regions of high bouton densities (termed as clusters). The boutons itself attributed to the five different clusters are

indicated with different colors (black, red, green, blue and yellow in order of their corresponding increasing cluster rank). Boutons outside clusters are marked in grey. (**d**) The top view of the axonal tree (grey) and the dendritic tree (black) are displayed together with the anterior-posterior axis, the vertical meridian and the neuron's preferred orientation. (**e**) The boutons (black) are densely packed in the neurons vicinity and are clotted at distant regions roughly 1 mm away from the soma. These high-density regions, nicely captured by the cluster-algorithm (**f**), are interleaved by sparse regions. Note that very few individual boutons and no superficial bouton clusters (black, red, blue, yellow) elongate along the neurons preferred orientation. (Neuron ID 11).

B. Six example superficial layer pyramidal neurons

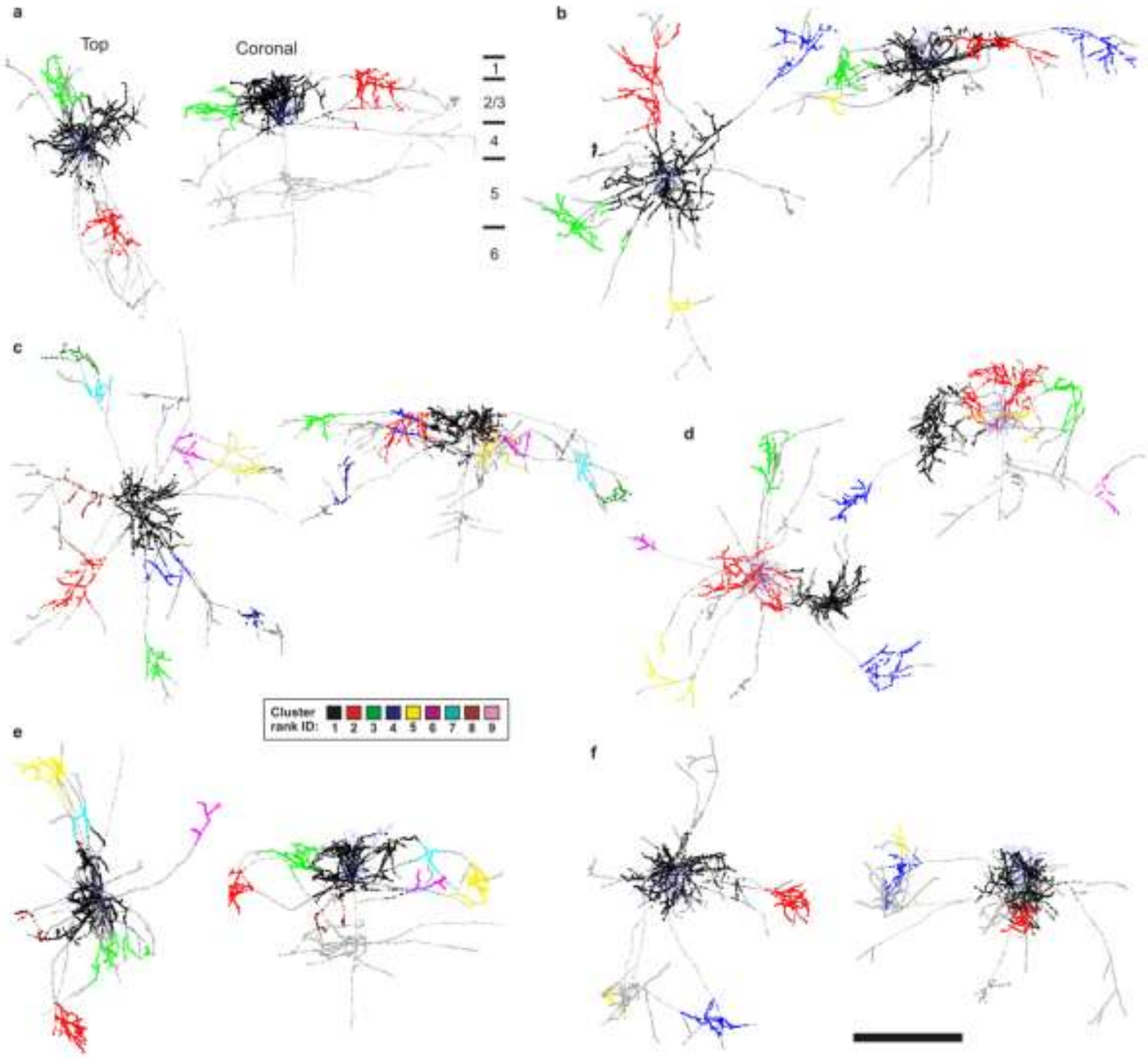

Supplementary Figure 2 Six examples of superficial layer pyramidal neurons in their top and coronal view. (**a-f**) Single neurons are displayed in top view (left) and coronal view (right, with layers indicated). Axons, non-clustered boutons and boutons of deep layer clusters are shown in grey. Clustered boutons are colored according to their rank (see color code). Scale bar=1mm. (Neuron IDs: **a (17)**, **b (7), c (13)**, **d (26)**, **e (20)**, **f (24)**).

C. Topview of the first 16 out of 33 superficial layer pyramidal neurons used in this study.

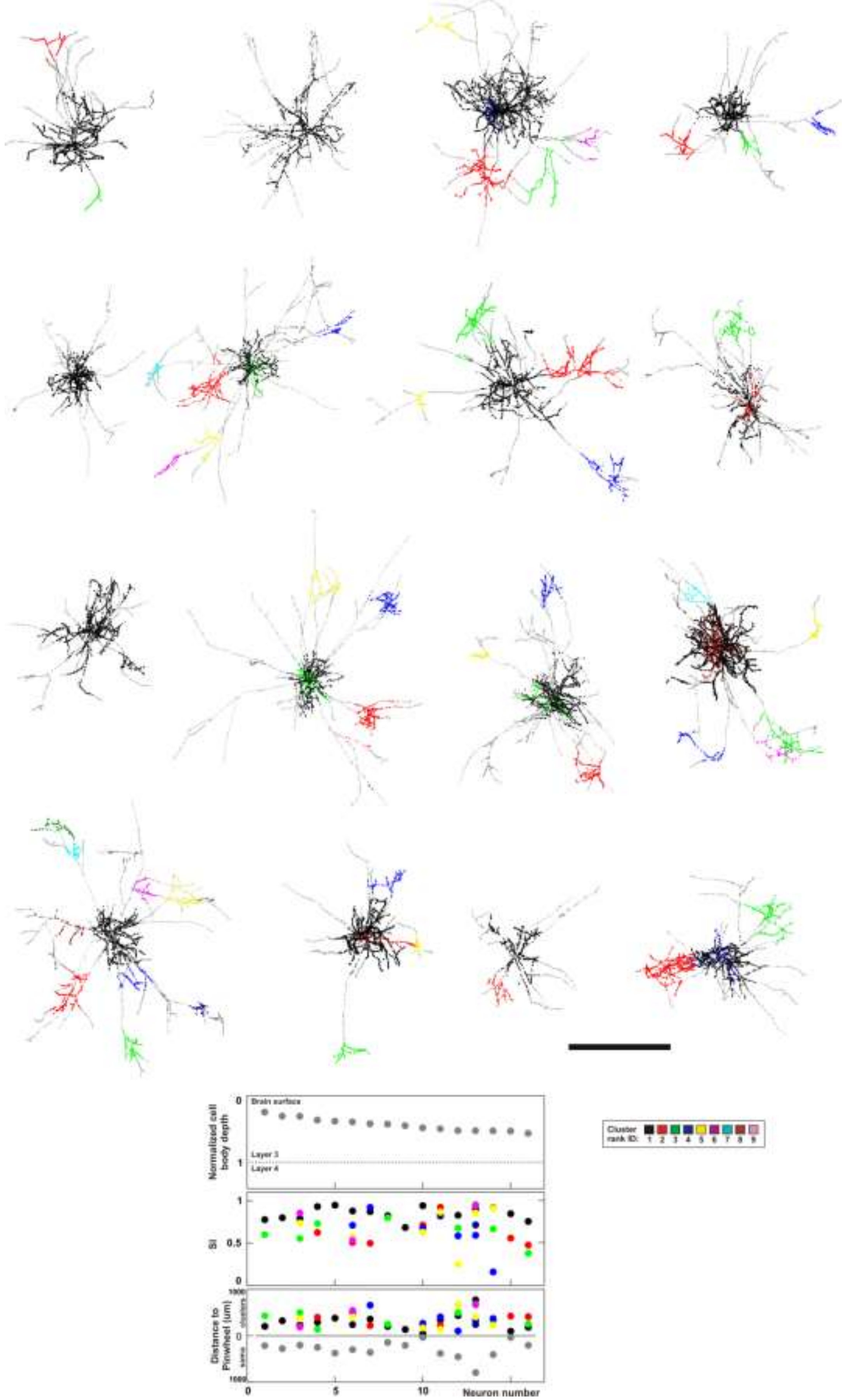

Supplementary Figure 3 Topview of the superficial layer pyramidal neurons used in this study. Shown are the first 16 neurons out of the 33 (for conventions see **Supplementary Figure 1**).

Neurons are sorted by normalized depth of soma. Each neuron was individually rotated that the space between was used best. Note that the neurons are shown in their entirety (incl. deep layer processes) whereas the actual study investigates only those parts in the superficial layers. Scale bar = 1mm. For reference and comparison are the graphs from **Figure 3** and **Figure 5g** shown at the bottom. Note that the number of dots at the bottom may be less than the actual clusters per neuron because for the analyses only superficial layer clusters within the mask were considered. The neuron IDs and their file name are as following: (1) Cat_0907_RH_axon_02.xml, (2) Cat_0408_LH_axon_02.xml, (3) Cat_1007_RH_axon_02.xml, (4) Cat_0907_RH_axon_01.xml, (5) Cat_2806_RH_axon_07.xml, (6) Cat_2606_LH_axon_05.xml, (7) Cat_0308_RH_axon_03.xml, (8) Cat_2806_RH_axon_03.xml, (9) Cat_1207_LH_axon_01.xml, (10) Cat_2806_RH_axon_06.xml, (11) Cat_1007_RH_axon_01.xml, (12) Cat_0608_RH_axon_02.xml, (13) Cat_0408_RH_axon_01.xml, (14) Cat_0608_RH_axon_03.xml, (15) Cat_2806_RH_axon_05.xml, (16) Cat_2806_RH_axon_04.xml.

D. Topview of the last 17 out of 33 superficial layer pyramidal neurons used in this study.

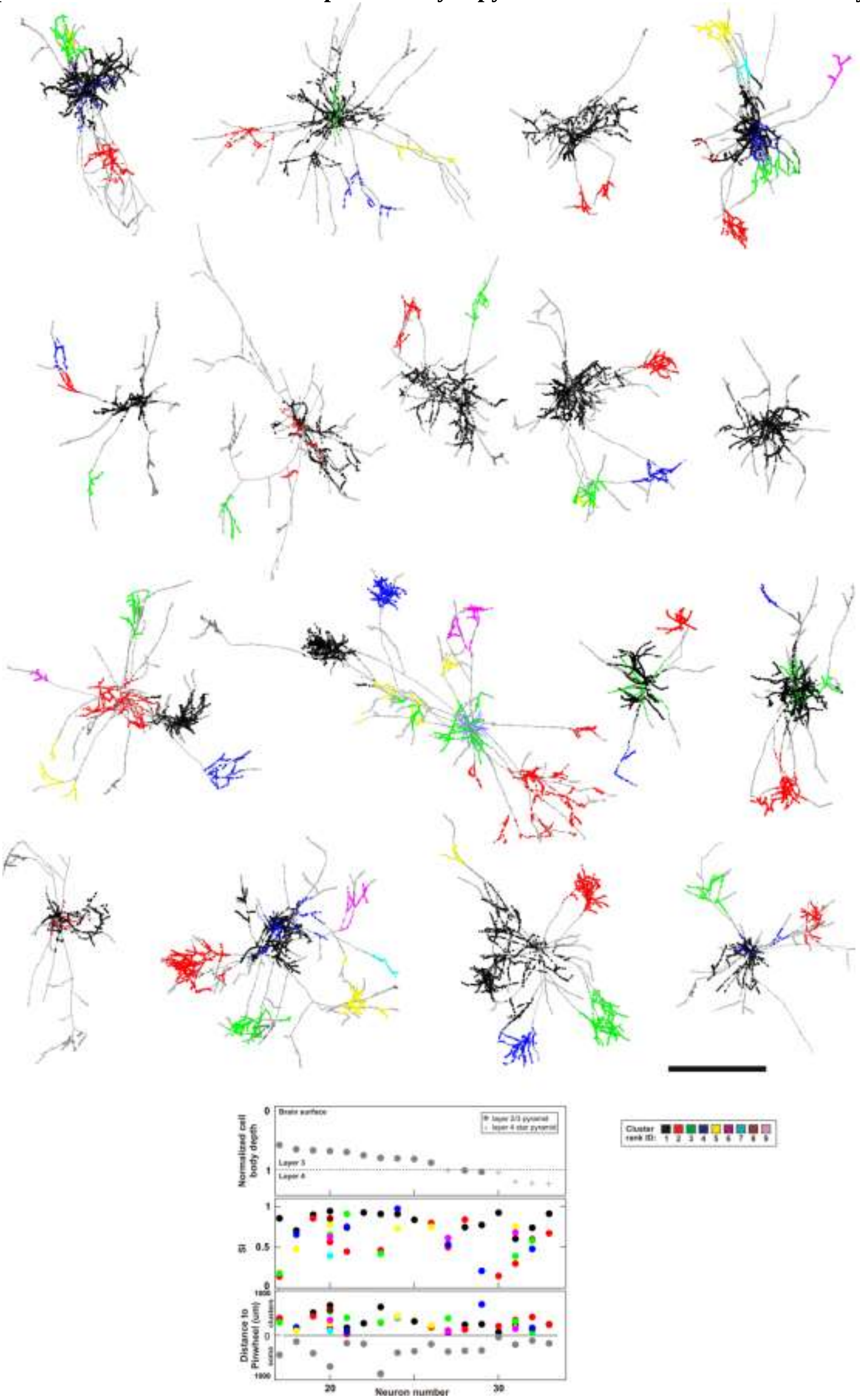

Supplementary Figure 4 Topview of the superficial layer pyramidal neurons used in this study. Shown are the last 17 neurons out of the 33 (for conventions see **Supplementary Figure 1**).

Neurons are sorted by normalized depth of soma. Each neuron was individually rotated that the space between was used best. Note that the neurons are shown in their entirety (incl. deep layer processes) whereas the actual study investigates only those parts in the superficial layers. Scale bar = 1mm. For reference and comparison are the graphs from **Figure 3** and **Figure 5g** shown at the bottom. Note that the number of dots at the bottom may be less than the actual clusters per neuron because for the analyses only superficial layer clusters within the mask were considered. The neuron IDs and their file name are as following: (17) Cat_0608_RH_axon_01.xml, (18) Cat_2806_RH_axon_01.xml, (19) Cat_0408_LH_axon_01.xml, (20) Cat_0608_RH_axon_06.xml, (21) Cat_1207_RH_axon_03.xml, (22) Cat_2606_LH_axon_03.xml, (23) Cat_0308_LH_axon_01.xml, (24) Cat_0707_RH_axon_01.xml, (25) Cat_0707_RH_axon_02.xml, (26) Cat_0507_LH_axon_01.xml, (27) Cat_0608_RH_axon_05.xml, (28) Cat_0807_RH_axon_02.xml, (29) Cat_0807_RH_axon_01.xml, (30) Cat_0108_RH_axon_01.xml, (31) Cat_0807_RH_axon_03.xml, (32) Cat_2506_RH_axon_01.xml, (33) Cat_0507_LH_axon_02.xml.

E. The neurons' individual sum-vectors.

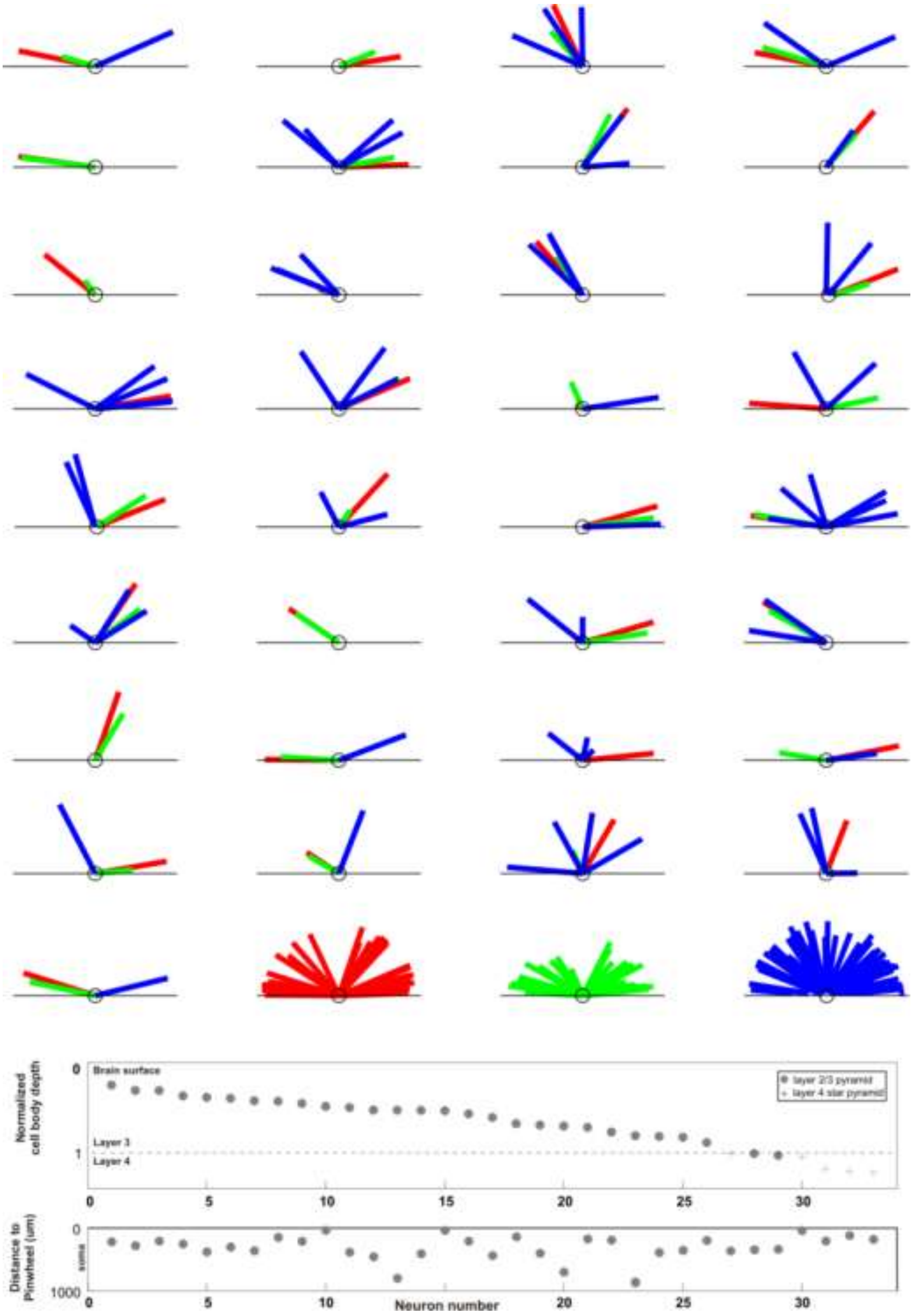

Supplementary Figure 5 The neurons' individual sum-vectors. Each neuron's sum-vectors are plotted as explained for two neurons in **Figure 2m, n**. Vectors in red represent the dendritic tree, vectors in green the local cluster and the blue vectors each one individual distal cluster (for details see **Fig. 2**). The 33 plots, i.e. neurons, were sorted by their depth from surface (see bottom). Note that the vectors for the local cluster (green) and the dendrite (red) point mostly in the same direction, i.e. similar orientation preferences, unlike the distal clusters (blue). Additionally, a neuron can have a differently tuned dendrite, local or distal clusters (see length of vectors). The last three plots are superpositions across all neurons to show that all angles are represented. For reference and comparison the graphs from **Figure 5f and g** are shown again at the bottom.

F. The first 16 out of 33 neurons and their orientation map

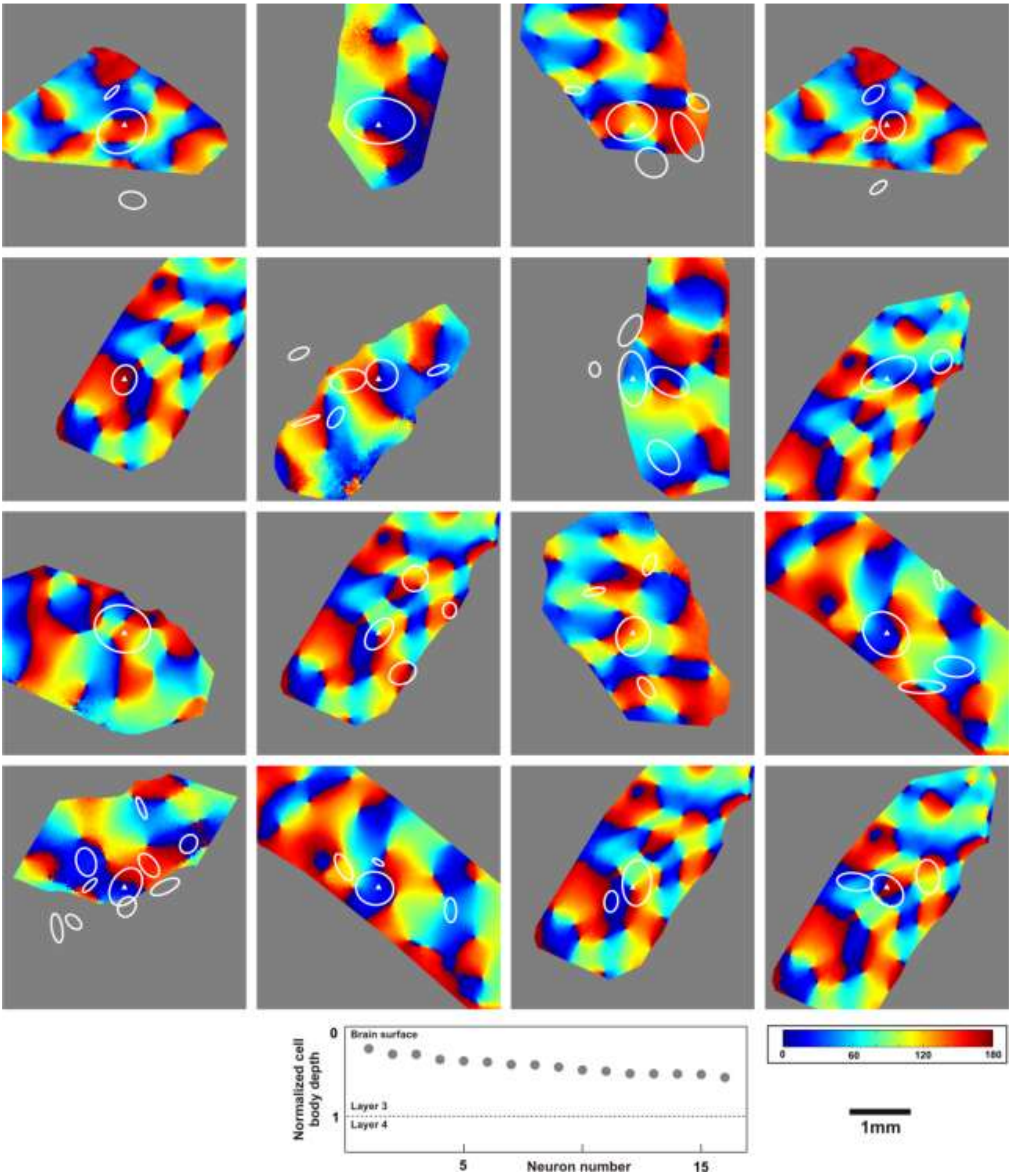

Supplementary Figure 6 Individual neurons overlaid on their corresponding orientation map. Neurons are represented only by their soma (white triangle) and their ellipses fitted to superficial layer bouton clusters (white curves). The first 16 neurons out of 33 are displayed, sorted by their normalized depth from surface as in **Figure 3**.

G. The last 17 out of 33 neurons and their orientation map

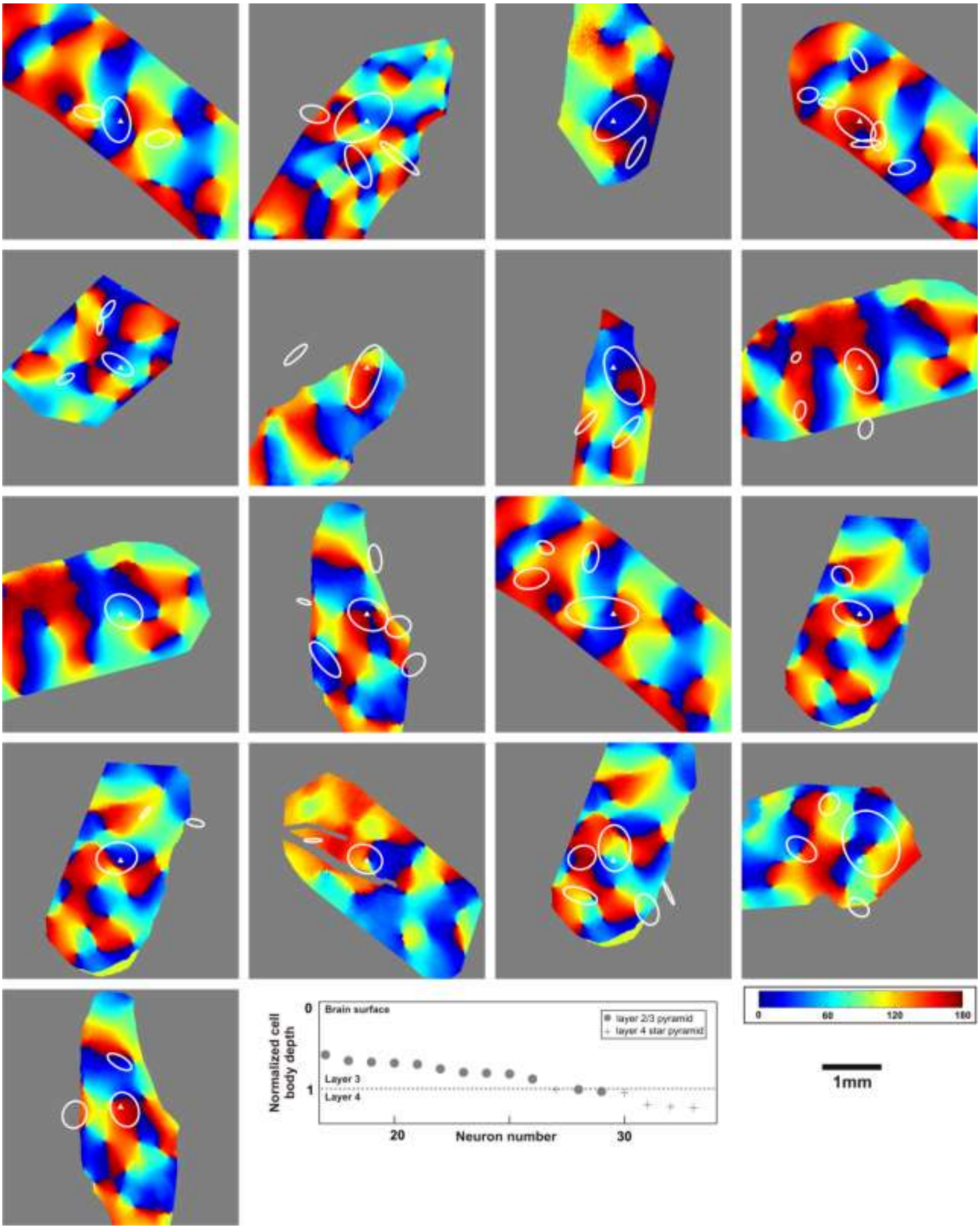

Supplementary Figure 7 Individual neurons overlaid on their corresponding orientation map. Neurons are represented only by their soma (white triangle) and their ellipses fitted to superficial layer bouton clusters (white curves). The last 17 neurons out of 33 are displayed, sorted by their normalized depth from surface as in **Figure 3**.

H. The neurons in their topview represented only by fitted ellipses to superficial layer bouton clusters.

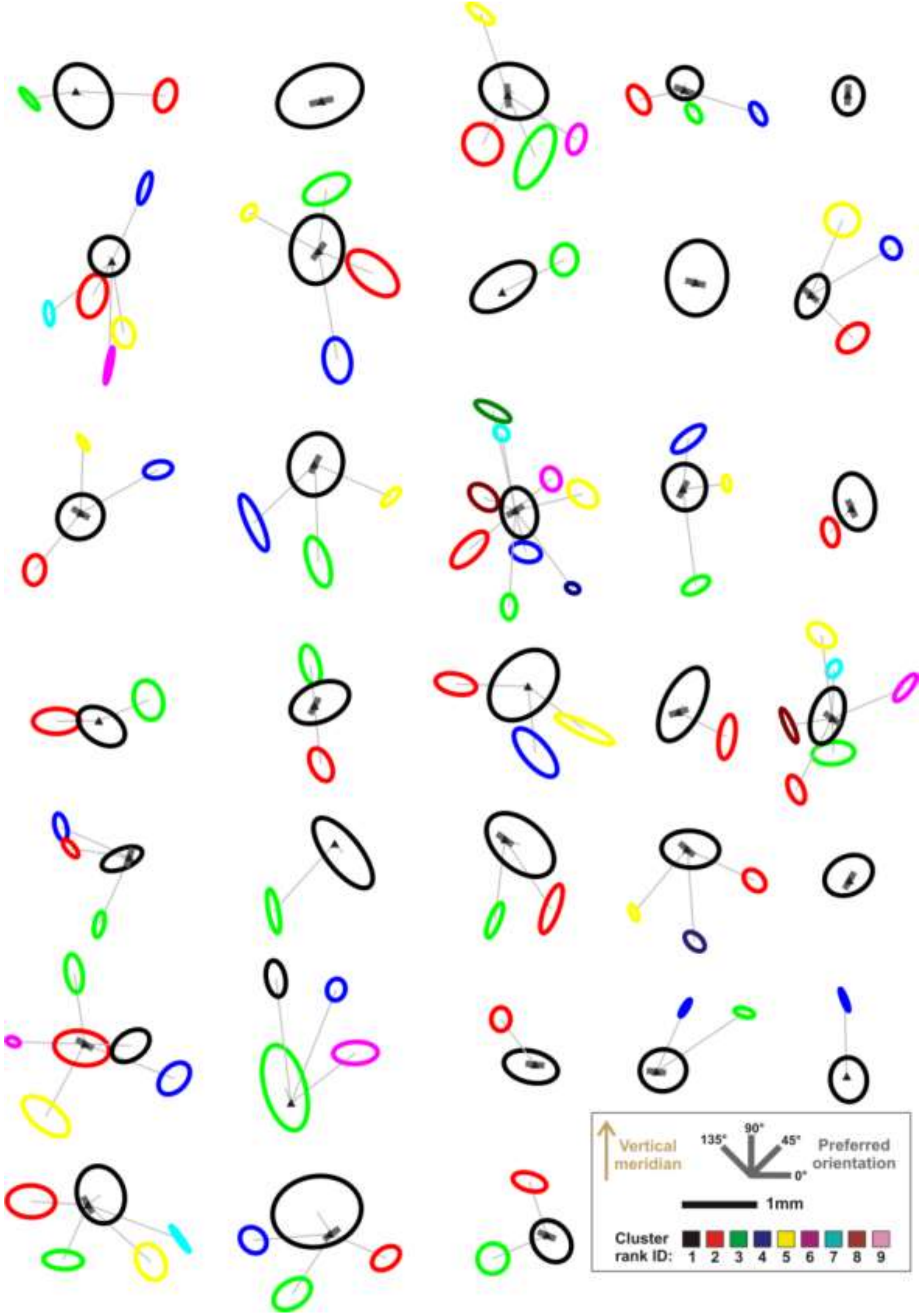

Supplementary Figure 8 The neurons in their topview represented only by fitted ellipses to superficial layer bouton clusters. All 33 neurons are sorted as in **Figure 3** and individually rotated to align with the vertical meridian (gold arrow). The soma (black triangle), the ellipses color-coded according to the cluster's rank (bottom right) and straight lines connecting each ellipse center with

the soma (grey lines) are displayed for each neuron. If available, the preferred orientation is plotted at the location of the soma matching the neuron's preferred orientation (bold dark grey bar).

I. Mean polar plots across 25 neurons with mapped Receptive Fields (RFs).

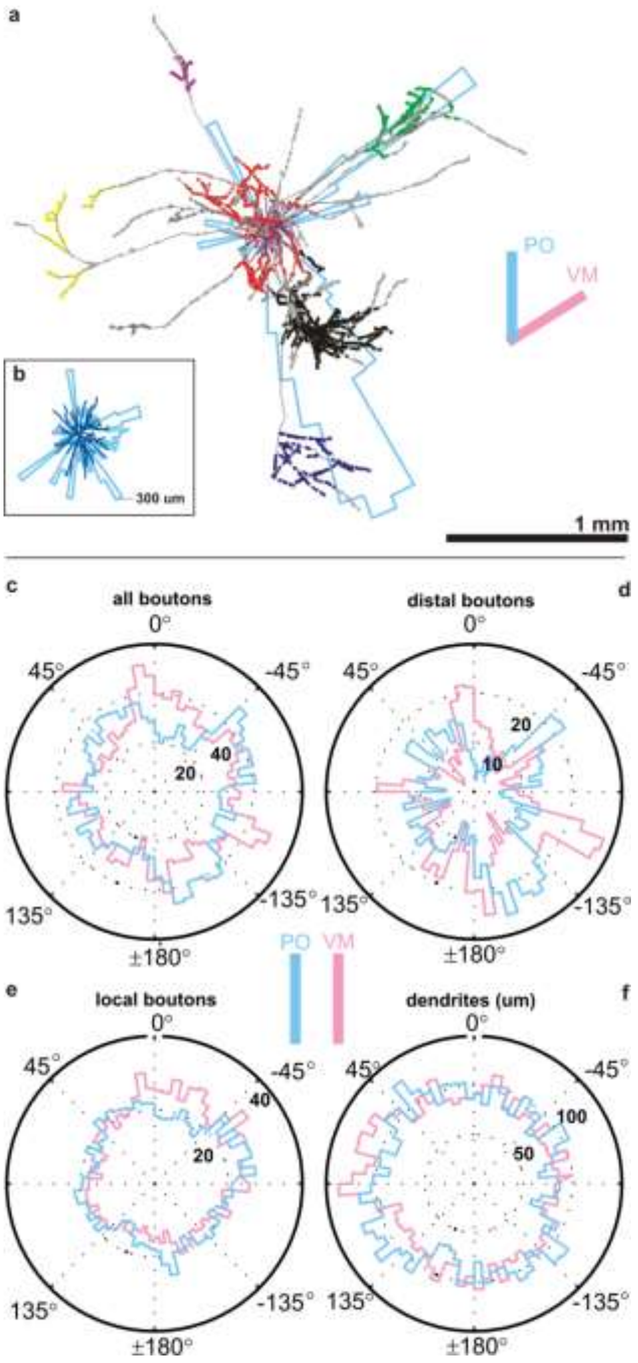

Supplementary Figure 9 Mean polar plots across 25 neurons with mapped RFs. Neurons were individually aligned to either the vertical meridian or the neuron's preferred orientation. (**a**) Example pyramidal neuron (ID: 26) shown in top view, rotated that the neuron's preferred orientation (blue bar) lies vertically. Pink bar indicates angle of vertical meridian representation. Boutons colour-coded black, red, green, blue, yellow in order of increasing cluster rank, grey boutons lie outside clusters. Beneath is a polar plot generated by counting total number of clustered boutons in 5 degree radial sectors (scale bar denotes 100 boutons in radial sectors). (**b**) The dendritic tree (blue) was aligned to the neurons preferred orientation and superimposed on a polar

plot in which the total dendritic length was calculated within each 5 degree sector. (**c-f**) Pooled polar plots across all neurons. Each individual neuron was either aligned to the preferred orientation (blue curves) or the vertical meridian (pink curves). (**c**) Mean polar plots of all clustered boutons for all neurons. (**d**) Mean polar plot of all distal clustered boutons. (**e**) Mean polar plots of boutons in local clusters. (**f**) Mean polar plots of all dendrites. No plot was significantly different from a circular distribution, i.e. no preference in any direction (Wilcoxon Sign Rank: **c**: p=0.59 / **d**: p=0.96 / **e**: p=0.75 / **f**: p=0.87). (Neuron ID: 26, cat_0507_LH_neuron_01. Simple RF, ocular dominance n.a., size 1.8x0.3°, location -2.6° / -5° from areal centralis, preferred orientation 150° ± 38°, direction preference 60°, 70 MΩ).

J. Step-by-step explanatory figure of the bootstrap procedure for a second neuron

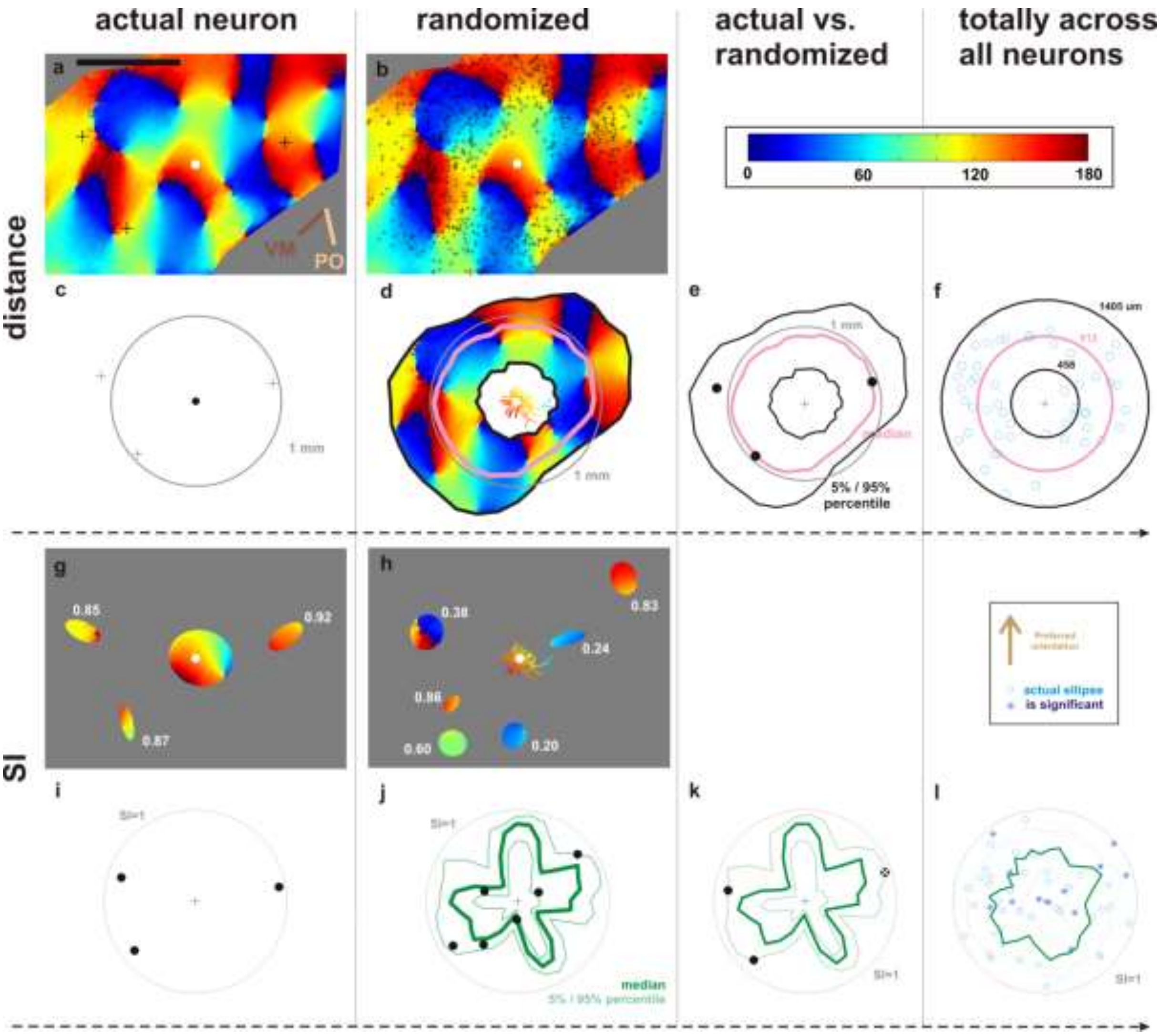

Supplementary Figure 10 Bootstrap of distal ellipses for a different neuron than the one shown in **Figure 8**. The figure is subdivided into four parts (‘*actual neuron*’, ‘*randomized*’, ‘*actual versus randomized*’ and ‘*totally across all neurons*’). The upper half, **a-f**, depicts the distances of the ellipses, the lower half, **g-l**, the SI values. (**a**) Displayed are the orientation map, the location of the soma (white dot) and the centers of the distal ellipses (pluses) (for details see **Fig. 1**). The neurons’ preferred orientation (gold bar) and the vertical meridian (brown bar) are marked at the bottom right. (**b**) Orientation map and the location of 1,000 bootstrapped distal ellipses out of the 20,000 that were generated. The size, distance from soma, shape and orientation of each individual bootstrapped ellipse was randomized based on the statistics of all distal ellipses totally across all

neurons (see Methods). (**c**) Displayed are the centers (pluses) of the three distal ellipses in relation to the soma (black dot). (**d**) Of all 20,000 bootstrapped ellipses those having an ellipse-center outside the orientation map or falling within the local ellipse were ignored. Of all the remaining ellipse centers (N=16,722) the radial distance to the soma was calculated. Instead of showing every single center, the median (pink contour), the 5% and the 95% percentile (black contours) were calculated and only those displayed (bin width = 10 degrees). The orientation map outside the percentiles was faded in white. Thus randomized ellipses are expected to occur on this displayed region of the orientation map. The dendritic tree was color-coded by its underlying pixels of the orientation map. (**e**) The three centers of the actual ellipses from **c** are shown as black dots. They are close to the median of all the bootstrapped ellipses (pink contour) signifying that a large number of the bootstrapped ellipses are located on top of the actual ellipses (compare crosses in **b** and **a**). (**f**) Superposition of all distal ellipses' centers (blue circles) totally across all neurons aligned to the neurons preferred orientation. The median distance (pink) and the 5 / 95% percentiles (black) determined the distribution of the radial dislocations from soma of the bootstrapped distal ellipses. (**g**) Pixels of the orientation map only within the fitted ellipses, the rest is faded in grey. The ellipses SI value is shown in white numbers (see **Fig. 2** for details). (**h**) Pixels of the orientation map only within the area of 6 bootstrapped ellipses out of the 1,000 in **b**. The rest of the map is faded in grey. The dendrite was color-coded by its underlying orientation map response values. For each individual bootstrapped ellipse its SI value was calculated (white numbers). (**i**) The ellipses of **g** are displayed at their correct circular location and as the radial distance their SI was taken. (**j**) The SI value was calculated for each bootstrapped ellipse and plotted in a radial manner originating at the soma. Instead of showing every bootstrapped ellipse's SI value, only the median (bold green), the 5% and the 95% percentile (light green) were calculated and displayed as circular plots (bin width of 10 degrees). The six bootstrap examples of **h** are shown as black dots. (**k**) The three actual distal ellipses from **i** are shown as black dots superimposed on the bootstrapped median (bold green) and percentiles (light green). If the actual ellipse had an SI value outside the statistically significant boundaries of the 5 and 95% percentiles they were termed as statistically significant (white cross through a black dot). One out of three actual clusters was statistically significant, highlighting that the actual ellipse was significantly outside the expected distribution of SI values in their particular sectors, even though many of the bootstrapped ellipses were actually located on top of the actual ellipses (see **e**, all three black dots are near the median). The other two ellipses were very close to the significant boundary. The percentiles for the significant test were determined based on all the bootstrapped ellipses within one sector (16,722/(36 sectors of 10 degree width)=464.5). (**l**) Superposition of all distal ellipses' centers (blue circles) totally across all neurons individually aligned to the neurons preferred orientation (unlike in **Figure 8e** where the neurons were aligned to the vertical meridian). Each dot has two coordinates: a circular and a radial one. The circular coordinates maintained the circular location in respect to the neuron's corresponding preferred orientation. The radial coordinates represent each clusters unique SI value as a radial distance from the center (black cross). A blue dot close to the grey circle (=1) signifies a high SI value. Each ellipse that was determined as statistically significant is marked with a purple cross (12 out of 51). To display all bootstrapped distal ellipses and their corresponding SI value totally across all 25 neurons, they were all superimposed and then the median (green bold) and the 5 / 95% percentiles (light green) generated and displayed. Obviously, the significant-test was on each individual neuron's percentiles performed and not on the here shown medians across all neurons. Note that the blue dots do not align along the preferred orientation, nor do the medians or the 5% and the 95% percentiles. Thus, distal ellipses can be encountered at all angles having high or low SI values. This holds on for the actual distal ellipses as well as for the bootstrapped distal ellipses.

K. Alignment of the 3D reconstructed brain tissue and in-vivo brain images.

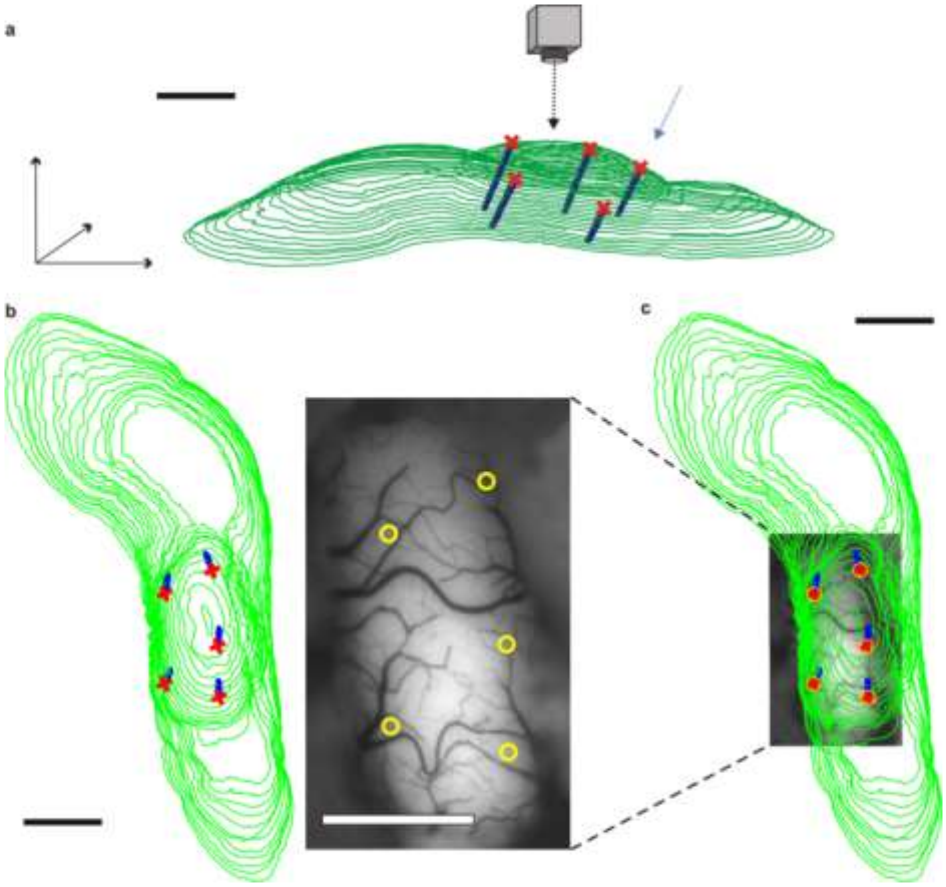

Supplementary Figure 11 Alignment of the 3D reconstructed brain tissue and in-vivo brain images. (**a**) Side view of the 3D reconstructed brain surface (green contour lines), the reference penetration tracks (blue) and their entry points into the brain tissue (red crosses). The penetrations were done vertically in reference to the stereotaxic coordinate (blue arrow). The camera was placed orthogonally (black arrow) to the brain region to obtain an evenly focused blood vessel-pattern as in **b**. (**b**) 3D contour lines, reference penetrations and their entry points. Next to it is the blood vessel-pattern and the locations of the reference penetrations (yellow circles) marked during the in-vivo imaging before the brain was processed. (**c**) The 3D reconstructions were overlaid with the linearly transformed in-vivo image. Scale bar = 2mm.

L. Key physiological parameters for all 33 neurons

ID	neuron name	Pref. Orient. (deg)	Range (+/-) (deg)	Direction (deg)	Distance between the soma to the next pinwheel (um)	Orientation of the angle map at the location of the soma (deg)	Orientation of the angle map at the location of the center of the local cluster (deg)
1	Cat_0907_RH_axon_02.xml	NaN	NaN	NaN	220	164	155
2	Cat_0408_LH_axon_02.xml	13	21	283	268	12	17
3	Cat_1007_RH_axon_02.xml	95	14	185	217	113	121
4	Cat_0907_RH_axon_01.xml	160	20	NaN	256	166	166
5	Cat_2806_RH_axon_07.xml	87	16	NaN	371	173	172
6	Cat_2606_LH_axon_05.xml	NaN	NaN	NaN	283	1	9
7	Cat_0308_RH_axon_03.xml	54	13	NaN	361	54	54
8	Cat_2806_RH_axon_03.xml	NaN	NaN	NaN	161	48	38
9	Cat_1207_LH_axon_01.xml	166	24	256	208	141	138
10	Cat_2806_RH_axon_06.xml	138	21	228	27	160	161
11	Cat_1007_RH_axon_01.xml	150	9	240	336	133	128
12	Cat_0608_RH_axon_02.xml	63[A]	47	153	454	21	22
13	Cat_0408_RH_axon_01.xml	31	NaN	301	782	11	8
14	Cat_0608_RH_axon_03.xml	63[A]	47	153	406	22	28
15	Cat_2806_RH_axon_05.xml	114	6	204	28	155	112
16	Cat_2806_RH_axon_04.xml	NaN	NaN	NaN	202	174	174
17	Cat_0608_RH_axon_01.xml	63[A]	47	153	434	19	28
18	Cat_2806_RH_axon_01.xml	NaN	NaN	NaN	141	49	33
19	Cat_0408_LH_axon_01.xml	17	15	287	387	16	19
20	Cat_0608_RH_axon_06.xml	144	20	234	688	173	178
21	Cat_1207_RH_axon_03.xml	72	11	342	183	54	34
22	Cat_2606_LH_axon_03.xml	NaN	NaN	NaN	181	151	160
23	Cat_0308_LH_axon_01.xml	143	8	233	870	15	177
24	Cat_0707_RH_axon_01.xml	138	17	228	390	145	143
25	Cat_0707_RH_axon_02.xml	60	NaN	NaN	340	74	63
26	Cat_0507_LH_axon_01.xml	150	38	60	190	2	179
27	Cat_0608_RH_axon_05.xml	NaN	NaN	NaN	397	5	38
28	Cat_0807_RH_axon_02.xml	174[B]	40	264	339	14	4
29	Cat_0807_RH_axon_01.xml	174[B]	40	264	332	14	12
30	Cat_0108_RH_axon_01.xml	NaN	NaN	NaN	44	141	148
31	Cat_0807_RH_axon_03.xml	120	30	30	194	63	106
32	Cat_2506_RH_axon_01.xml	36	NaN	NaN	121	62	4
33	Cat_0507_LH_axon_02.xml	158	32	68	173	163	164

Supplementary table 1: Key physiological parameters for all 33 neurons. The neuron ID is given together with the name of the neuron, its key physiological parameters, the distance between the soma to the next pinwheel (um), the orientation of the angle map at the location of the soma and the orientation of the angle map at the location of the center of the local cluster. For some neurons we were not able to acquire all physiological parameters (NaN). The neurons marked with the letter A and B compose a triple and a pair respectively, which made the allocation of the physiology to one neuron not possible. At position A, the pipette tip got damaged after filling the cell and resulted in staining of more than one cell. At position B, while penetrating the target cell, the applied current pulse caused damage to a second cell and thus two cells got filled simultaneously.

M. Functional specificity of long-range horizontal connections

Publication	Species	Area	Functional modality	Preference (%)
**Malach et al. (1993)**	Macaque monkey	V1	Ocular dominance	65
**Malach et al. (1993)**	Macaque monkey	V1	Orientation	66
**Malach et al. (1994)**	Squirrel monkey	V2	Orientation	50.3
**Yoshioka et al. (1996)**	Macaque monkey	V1	Cytochrome Oxidase - blob	68
**Yoshioka et al. (1996)**	Macaque monkey	V1	Cytochrome Oxidase -interblob	73
**Yoshioka et al. (1996)**	Macaque monkey	V1	Ocular dominance	63
**Kisvarday et al. (1997)**	Cat	A17	Orientation	53
**Bosking et al. (1997)**	Tree shrew	V1	Orientation	57.6
**Schmidt et al. (1997a)**	Cat	A17	Orientation	58
**Schmidt et al. (1997b)**	Cat	A18	Ocular dominance	56

Supplementary table 2: The functional specificity of long-range horizontal connections matches on average 61% the functional specificity of the origin of the projection. These investigations used exclusively bulk-injections, hence labeling dozens to hundreds of neurons in an antero- and retrograde fashion and labeling a mixture of both excitatory and inhibitory neurons.

Supplementary Methods: Description of the NEREDA framework

Introduction

Nereda is an open source framework to analyze neuronal reconstruction data. The software is a Java/Matlab hybrid that uses the speed of Java and the flexibility of Matlab code. The architecture makes it accessible from both sides, i.e. from within Java and from within Matlab script code, even though the Java approach with its strong IDEs, e.g. Eclipse, is the recommended way to use it.

Jobs can be started by using NeredaCLI (Nereda does not include a rich GUI client). The user has to create project specific packages and analysis classes. The target group are scientists with at least basic knowledge of Java, who want to use a flexible, robust and maintainable architecture to analyse and visualize their data.

Matlab analysis code can be wrapped into Java classes to be used in Nereda. Additional data, e.g. cell types and physiology, may be added and used in the analysis as well. The core classes provide means to save analysis results as Matlab data files, and to visualize data in Matlab figures. They can be used in publications or for further analysis steps.

You may wonder why Nereda has to be compiled for each platform separately, although it's based on Java technology. This is due to the Matlab Compiler Runtime (MCR), where Nereda's and your m-scripts are executed. MCR unfortunately is NOT platform independent. Nereda's m-scripts and Java source code however are. You should be able to compile it on your platform without any changes.

The simplest way to use Nereda, is to download the VirtualBox appliance with preconfigured Eclipse IDE and program project specific source codes for desired analysis. Enough RAM on your host system, at least 6GB recommended.

Start VirtualBox, and select Import Appliance... from the File menu, and select the ovf file you just downloaded. Adjust the import settings (RAM...) in the intital dialog of VirtualBox, and import the image. The import can take some time. Start Eclipse from the Desktop.

Philosophy of the framework

The framework was designed to work in a very dynamic environment with changing and sometimes conflicting requirements, i.e. quantitative brain research. For not losing the robustness and maintainability of the software package after a short time, the framework is designed along specialized guidelines. First the data acquisition in 3D reconstruction modules and the data analysis and visualization in *Nereda* are completely separated. *Nereda* never changes the original data or structures gathered in the reconstruction software. This allows collaboration on stable data sets, and a revalidation of the original data at any point in the analysis process (***Figure A.1***). Second *Nereda* keeps a rigid separation of data on one side and the data processing logic on the other side. All the processing logic is encapsulated in visitor classes which implement a simple interface and exist independent of the hierarchical data structure. The framework can easily be extended in a robust manner by adding additional visitors without compromising existing code or having to take care about the underlying data pool. Third, the consequent interface based programming in the whole framework provides an easy and well-defined mechanism to extend the packages, either on the analysis or more fundamental on the data object representations, which opens the framework for other data pools beside of *Neurolucida*™. Fourth, *Nereda* can be accessed in a very flexible platform independent way from Windows-, Linux- or MacOS environments, from within both *Java*- and *Matlab* programming environments. By using the *Java* programming language the framework is not only platform independent and fully object oriented, but it also outperforms the speed of pure *Matlab* implementations by orders of magnitude.

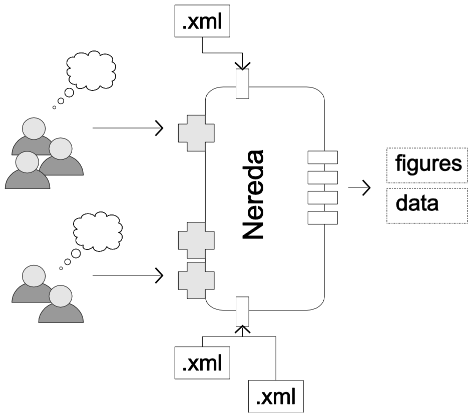

Figure A.1: *Nereda* workflow: Several researchers or groups of researchers develop their own ideas how to analyze chunks of reconstruction data (data provided as *.XML*). Each group implements their analysis as series of new *Nereda* plugins or uses the plugins of other groups or built-in ones. Several of these components can be merged to super-plugins. The framework guarantees the interoperability of the components.

The mentioned implementation principles allow keeping the robustness of the framework even if the requirements change rapidly or when several programmers or scientists extend the packages by their own components. The result is a flexible and compact software framework to analyze and visualize neuronal reconstruction data, which keeps its robustness and maintainability over time.

Architecture

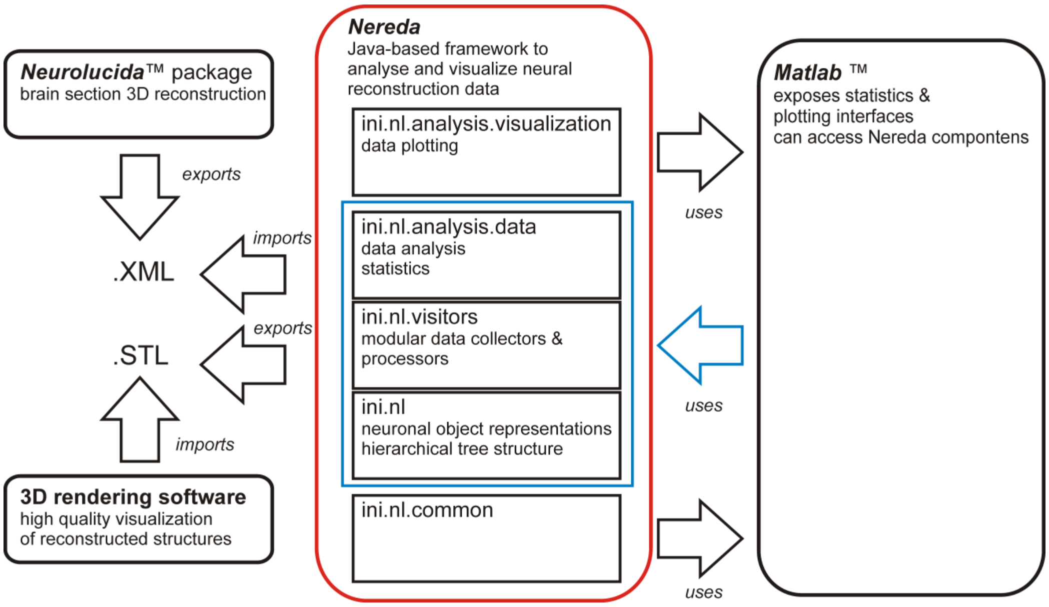

Figure A.2: The *Nereda* architecture: *Nereda* resides in the middle between neural reconstruction packages, i.e. *Neurolucida*™, the data processing language *Matlab* and 3D rendering software packages like *Blender*. *Nereda* is a software framework written in *Java* that encapsulates the complexity of data structures and internal processing logics. The framework provides simple interfaces to plug-in custom analysis code. Several namespaces contain built-in components for data collection, statistics, and visualization. Interfaces to *Matlab* (*Java*-based interfaces) and render software packages (*STL*, Surface Triangulation Language) provide full flexibility for future requirements.

Nereda positions itself in the middle between the data analysis environment *Matlab* and 3D reconstruction software packages, e.g. *Neurolucida* and 3D rendering software solutions (**Figure A.2**). The framework imports neuronal 3D reconstructions via *XML* data structures to build up a *Java* based object hierarchy. Each instance in the hierarchic tree represents a member of object-oriented class architecture that implements well defined interfaces (*ini.nl*-namespace, *ini.nl.inf*-namespace). Any of the hierarchy elements, e.g. cell soma, axonal/dendritic segment and varicosity, deserves as an entry point for worker classes (*visitors*) that fulfill various tasks, e.g. data collection, statistical analysis and plotting (*ini.nl.visitors*-namespace). The object tree itself guarantees that the worker instance starting from the entry point visits and executes its code for all of the tree sub-elements. This leads to an optimal separation between code logics and tree internal data organization, i.e. optimal robustness with the most flexibility. Predefined libraries for data analysis and visualization provide easy access to statistics and ad-hoc plots (*ini.nl.analysis.data*- and *ini.nl.analysis.visualization*-namespaces). In addition the interface based approach allows easy extension of the framework parts, while maintaining its robustness. The analysis components of *Nereda* have complete access to *Matlab* data processing procedures. In turn most of the *Nereda* components are accessible from both *Java* and *Matlab* to provide the best freedom of choice for *Nereda* programmers. For high quality visualizations *Nereda* provides functions to export fractions or complete neurons and brain surfaces into the Surface Triangulation Language (*STL*) used in stereo lithographic CAD- and rendering packages like *AutoCAD*™ or *Blender*. In addition *Nereda* allows the saving of complete object trees into the *Matlab* proprietary .mat-format for easy exchange between users or computers.

Brain domain model

The core of the framework is represented by the *ini.nl*-namespace classes. Anatomical real-world entities, e.g. Neuron, Axon, Dendrite etc., are modeled by classes from this namespace. *Nereda* represents the reconstructed data as a hierarchical object tree rooted in the *Brain* entity (***Figure A.3***). A brain contains a series of section outlines which define its spatial borders. In addition the brain object can hold neurons and a number of reference penetrations that deserve as landmarks. To relate the anatomical data to optical imaging a collection of optical maps can be attached to the brain. Each component in the hierarchy that has a specific location or spatial extent is defined as a series of points in x/y/z-space with an optional diameter d.

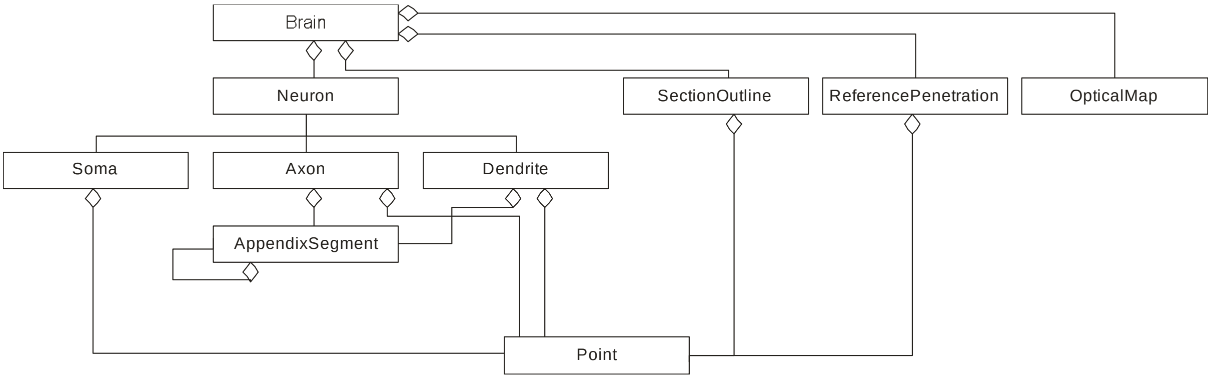

Figure A.3: Simplified *Nereda* brain domain model: The reconstructed brain structures, i.e. brain surface and neuronal compartments, are modeled with a series of related objects. To keep the application of the framework flexible all relations between objects are modeled as aggregations, rather than associations (each component can exist isolated from its parent object). Four main components, i.e. Neuron, OpticalMap and ReferencePenetration all rooted in the Brain object build the backbone of the hierarchy. Together with their subcomponents they form a complete, sequentially accessible object tree, which represents the reconstructed *Neurolucida*™ data.

Traversing the object tree

Nereda uses data collected in *Neurolucida*™ to construct a hierarchical object structure. The building blocks of the object tree are located in the *ini.nl*-namespace. Once the tree structure is initialized it can be traversed either sequentially or via random-access (***Figure A.4***). Sequential access is provided by typed collections. Since each tree element, beside of the root has exactly one parent object, each tree element and associated data is accessible via a unique path. However, to keep the framework as flexible as possible the relationships between the classes are all modeled as aggregation, rather than compositions, i.e. each element can exist without a parent object. Random access is provided at each tree node via a search-for-element-id mechanism provided via the *getElementById(String id)*-method. More sophisticated filters can easily be added via custom visitor classes.

A third method to traverse the tree, i.e. *visitor-based traversing* is described in the next section. Visitors also sequentially access the tree. The explicit logics how to perform the walk in the complex heterogeneous tree structure is however hidden from the programmer, and replaced by a simple but powerful interface.

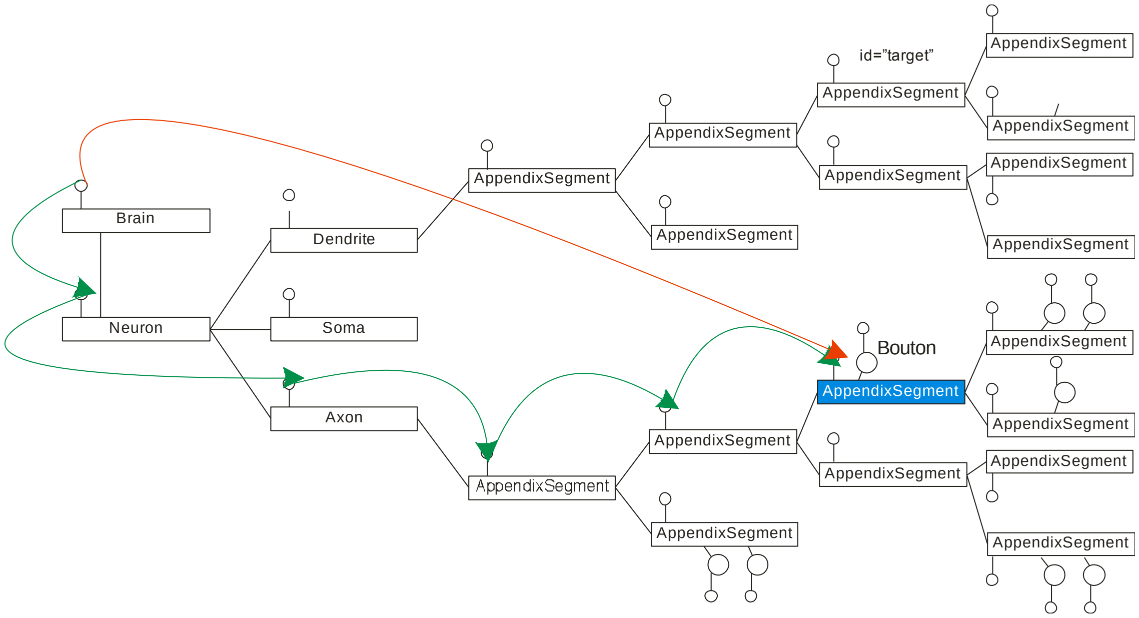

Figure A.4: Schematic, simplified tree structure that represents a brain with one neuron: The figure shows a simplified *Nereda* tree structure to illustrate access modes in *Nereda*. Boxes represent elements/nodes of the tree, large circles boutons on axonal tree segments, and small boutons interfaces exposed by the elements. *Nereda* provides two access modes, i.e. sequential and random. In sequential mode one *walks* element-by-element through the tree to access the target element. In random access internal *Nereda* search functionality allows direct access to the target from any parent element in the tree.

Collecting/processing data

The most easy and flexible way to collect or process data in *Nereda* data structures is to use visitors.

The visitor pattern

Nereda encapsulates internal data structure logics and hides it from the user. The programmer does not have to know the exact structure of the internal object tree, or how to traverse this arbor to collect or process data. In the visitor pattern one distinguishes between the visitor (the component that walks through the tree) and the visited object. In *Nereda*, any tree element can be a *visited object*, i.e. each element in the tree has to implement the *VisitedInf-interface* which allows injecting a visitor. The visitor on the other hand implements the *VisitorInf*-interface, which defines two methods, i.e. *execVisit* and *execAfterVisit*, that are called from the visited element when the visitor arrives at the instance at his way down through the tree and on his way back, when it arrives a second time at the same element. The visitor component automatically *visits* the sub-elements of the entry point to execute its code (***Figure A.5***). This strategy which hides internal tree logics from the programmer is optimized to deal with varying heterogeneous hierarchical structures, e.g. neurons in brains. The visit-procedure comes in two flavors, i.e. a shallow one where only the direct sub-elements of the entry point are considered (*visit*), and a deep visit variant where all the elements of the sub-tree rooted at the entry point are traversed (*visitDeep*). The interface-based separation of internal data structure and data processing code makes the framework robust and flexible at the same time.

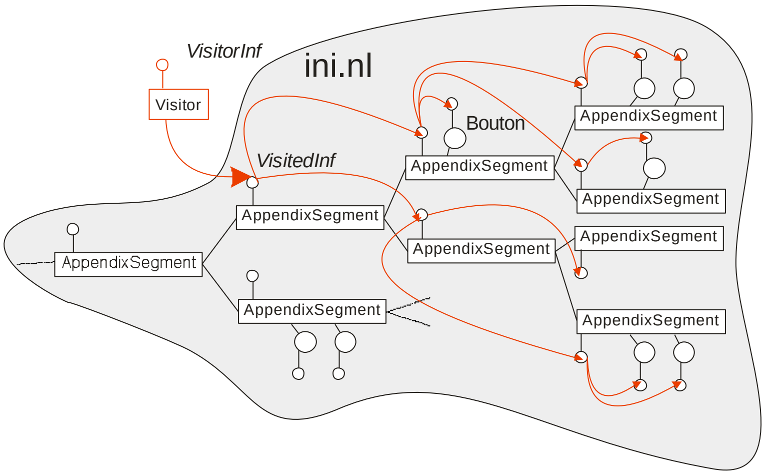

Figure A.5: Visitor pattern in a deep variant: A customized data processing component (Visitor) is injected into a heterogeneous hierarchical tree. The component automatically travels through the sub-tree and visits each of its elements to execute its customized code. Any element in the tree can deserve as an entry point for the visitor.

Nereda Concepts

This chapter gives an overview on a number of important aspects of Nereda, and illuminates how the framework was built. There are often much more elegant solutions to a problem if one keeps in mind the object oriented architecture.

Projects

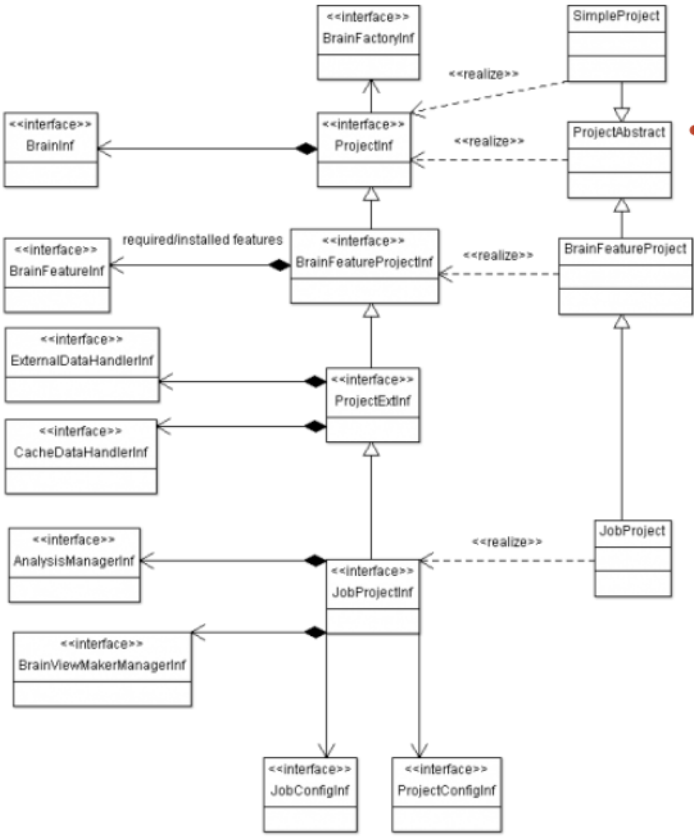

Figure A.6: Nereda project interfaces and their realizations

Depending on the context a series of project types are provided. In all cases the project holds a list of brains, whereas each brain represents the root of an object tree, containing neurons, analysis objects, view makers, etc. The brains are created by brain factories based on reconstruction content, optical imaging maps and additional data resources.

Amongst the object types the simplest one, intended to be used for ad-hoc projects, is SimpleProject. This type has a minimal set of configuration overhead, and all project components, e.g. brain, neuron, analysis, have to be instantiated manually, e.g. by virtue of a brain factory.

The next type BrainFeatureProject in contrast to the simple implementation provides means to use Nereda brain features. Brain features provide a consistent mechanism to extend reconstructed content, with additional data, e.g. in which hemisphere we are, layer surfaces, layer membership of items, cell type information and so on. The brain features have to created and attached to the project in your code.

A JobProject is the type of project used by the NeredaJobRunner. It has configuration objects attached to itself, which register external data handlers (EDH) and cache data handlers (CDH). In addition this project type has a list of analysis managers and brain view maker managers, which deal with analysis execution and saving the results, and with creating brain visualizations that go beyond the standard

plotting routines. All components are configurable in XML-based settings files. By virtue of NeredaJobRunner a job can be run as a configurable standalone application.

Brains

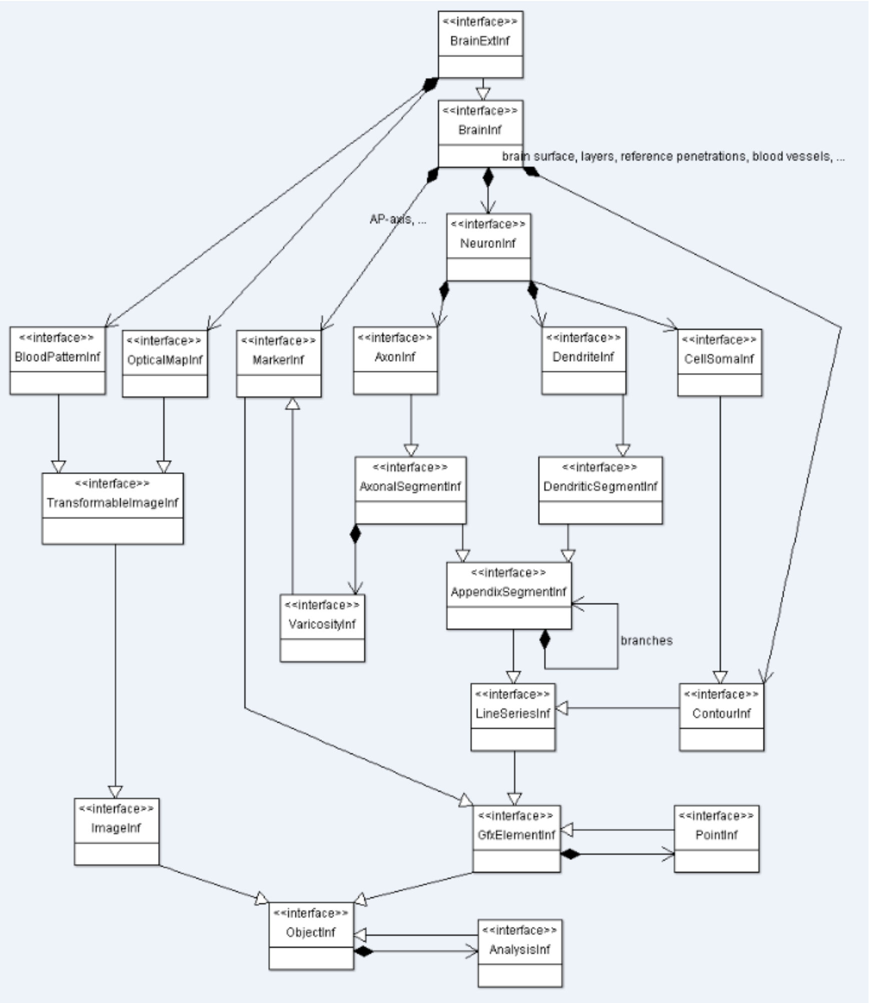

Figure A.7: Simplified view on Nereda brain interfaces

A brain object is the root of an object tree that was created based on reconstruction data (most often by virtue of a brain factory). A brain class at least implements *BrainInf*, and contains lists of contours that provide information about brain compartment boundaries, i.e. brain surface and layers, plus reference penetrations or blood vessels that may exist. In addition each brain can contain neurons (*NeuronInf*), each of which contains a list of axons and dendrites. Lists where chosen to be able to hold an incomplete representation of an axon or dendrite, given as independent sub-trees. Each of the axonal- or dendritic segments contains a list of sub-branches (zero if it represents an end-segment, two if we are in the middle of a tree). An axonal segment contains a list of varicosities, i.e. potential synapses, defined by their coordinates and thickness. Technically the segments are given as line series defined by a set of ordered points with thickness information. Any object in the tree that is defined by a set of points, whether a line series or not, implements *GfxElementInf*, which in turn is derived from *ObjectInf*, i.e. the base interface of any object in the hierarchy. Each object in addition implements *VisitedInf* and can therefore be target of a visitor that traverses the tree. In addition each object can act as an analysis target.

Beside of the base brain interface *BrainInf*, *BrainExtInf* exists which declares accessors for optical imaging maps (single condition orientation maps) and blood pattern images. The interfaces were

separated since the most common input to Nereda, i.e. Neurolucida reconstruction data, does not include non-anatomical data. The images/maps provide functions to register neuronal reconstructions and image data in 3-D space.

The picture shows only a simplified view of the hierarchy. To get a detailed view of all interfaces and classes the reader should refer to the javadoc of Nereda or to the source code.

Brain Factories

Nereda constructs brains by virtue of input data format specific brain factories that implement *BrainFactoryInf*. Each project has a brain factory attached. In JobProjects the factory is set in the project configuration file. The resources (input files with reconstructions, maps, ...) are given as file locations. The factory decides based on the file type, the file properties and the content how to load the resources. The supported resources depend on the factory implementation. Currently the Neurolucida factory covers the types: Neurons, contours (brain surface, layers, reference penetrations, ...), single condition maps, map masks and blood pattern images. Each of the types need to be available as a separate file.

Brain Component Factories

A brain component factory implements *BrainComponentsFactoryInf* and provides methods to construct all required brain components, e.g. axonal segments, dendritic segments, boutons, etc. The component factory is input format independent, and is normally used by the format dependent brain factory to create object stubs which are filled up with data. The separation between brain- and brain component factory makes it possible to change the properties of a given object class for all input types with just a single factory class. Consider the following example: You want that all your neurons show their axonal/dendritic end segments in red, irrespective of the source of the reconstruction data (Neurolucida). To do this you just have to inherit from the given *BrainComponentsFactory* class and overwrite the *createAppendixSegment* method. Under assumption that the brain factory implementers followed the rule to create all brain components by virtue of the brain components factory, you are done now, all end segments in all neurons will be shown on red, irrespective in which application they were reconstructed. You use the new components factory by using the *setBrainComponentsFactory(BrainComponentsFactoryInf brainComponentsFactory)* method of the brain factory.

The code for the new *BrainComponentsFactoryForRedEndSegments* would look something like that:

```
private class BrainComponentsFactoryForRedEndSegmentsextends BrainComponentsFactory
implements BrainComponentsFactoryInf {
  public BrainComponentsFactoryForRedEndSegments() {
  }
  @Override
  public AppendixSegmentInf createAppendixSegment(String id, String name) {
    AppendixSegmentInf as = super.createAppendixSegment(id, name);
    if(as.getSegments().size==0){
      as.getPlotProperties().put("Color", new double[]{1.0, 0.0, 0.0});
    }
    return as;
  }
}
```

Brain Features

Brain features represent a single quality of information that in the sum make up a reconstructed brain. The most often used brain feature is *BrainFeatureReconstructionContent*, which by virtue of a brain factory and a list of resources creates and returns the brain object tree. The information in the tree can be extended by additional features, e.g. cell type information, preferred orientation, cell body location,

pinwheel locations on angle maps, etc. Brain features can depend on other features. *BrainFeatureCellType* for example depends on *BrainFeatureReconstructionContent*, since there is no target for a cell type if there are no cells in the brain. Nereda resolves these dependencies automatically in a recursive way and constructs an ordered feature list, which is processed to create the brain. However, for this to work a user of the framework has to register a complete list of features on the project (see *BranFeatureProject*). It is recommended to have a central method, that returns a complete list of all available features. In *JobProjects* the list can be configured in the project configuration file.

Beside of dependencies on other brain features, a feature can also depend on external data, e.g. a file that carries a lookup table that assigns a cell type to each reconstructed neuron. External data is handled by external data handlers (EDH).

External Data Handlers (EDH)

External handlers allow for access of external information to make it available to the Nereda framework. Currently these are often only comma separated value (csv) files which contain additional information collected during an experiment that is important for the analysis of the reconstructed neurons. An example for a csv file, which assigns a presentation angle to a single condition map is given below.

```
# Neuron physiology lookup table for the Nereda framework.
# Use the following numbers for each neuron.
# <Resource File Name> is the name of the brain xml file
# <singleMaps>, the filename of the single map (has to correspond to entries in
<Resource File Name>)

# Cat_0608_RH
src/resources/neurolucida/cat_0608/SingleMap_1.bmp, 0.0
src/resources/neurolucida/cat_0608/SingleMap_2.bmp, 22.5
src/resources/neurolucida/cat_0608/SingleMap_3.bmp, 45.0
src/resources/neurolucida/cat_0608/SingleMap_4.bmp, 67.5
src/resources/neurolucida/cat_0608/SingleMap_5.bmp, 90.0
src/resources/neurolucida/cat_0608/SingleMap_6.bmp, 112.5
src/resources/neurolucida/cat_0608/SingleMap_7.bmp, 135.0
src/resources/neurolucida/cat_0608/SingleMap_8.bmp, 157.5
```

The data source and the type of data that is retrieved depends completely on the external data handler. Some handlers are also capable to create external data. Whether or not a given handler supports data creation is accessible over the `canCreateData()` flag. External data handlers can depend on brain features, and analog to the brain features the handlers have to be registered on a project. Nereda automatically includes the handler features dependencies in the calculation of an ordered feature set.

Cache Data Handlers

Produce cache data which can be deployed together with the Nereda jar file to speed up execution time and reduce memory requirements.

Project Configuration File

Configurable Nereda projects, e.g. JobProjects are associated to a project configuration file that sets the environment variables. A simple project configuration file could look like this:

```
<?xml version="1.0" encoding="utf-8"?>
<nereda>
 <project>
   <externalDataHandlers>
     <description>Global external data handlers</description>
   </externalDataHandlers>
```

```
   <brainFeatures>
     <description>Brain feature registry with all features used in this
project</description>
     <feature name="BrainFeatureReconstructionContent"
className="ini.nereda.feature.BrainFeatureReconstructionContent"></feature>
   </brainFeatures>
   <params>
     <param name="externalDataPath" description="Root folder of the external data (csv-
files, ...)." value="./persistence" />
     <param name="cacheDataPath" description="Root folder of the cache data (zip-files,
...)." value="./cache"></param>
     <param name="tempDataPath" description="Root folder of the temp data (tmp-file,
...)." value="./tmp" />
     <param name="brainFactoryClassName" description="Brain factory to use"
value="ini.nereda.factory.BrainFactoryNeurolucida" />
     <param name="brainComponentsFactoryClassName" description="Brain components
factory to use" value="ini.nereda.factory.BrainComponentsFactory" />
   </params>
   <cacheDataHandlers>
      <description>Cache data handlers</description>
   </cacheDataHandlers>
 </project>
</nereda>
```

The project node has the sections *externalDataHandlers*, *brainFeatures* and *cacheDataHandlers*. These sections represent registries for features and handlers that might be used by analysis classes or other components. In this simple project we only use a single brain feature, the other sections are empty. Beside of that we have to set the mandatory parameters in the *param* section. For a more complex project file consider.

Job Configuration File

Beside of a project configuration a *JobProject* is also associated to a job configuration file, that contains all necessary information to run a job. The exact content depends on the job you run. A simple example could look the following:

```
<?xml version="1.0" encoding="utf-8"?>
<nereda>
 <jobs>
   <description>Nereda job configurations.  </description>
   <job id="Tutorial3" name="Tutorial3" description="Tutorial3 description"
type="Analysis" outputUrl=".\output\result\analysis"
logUrl=".\output\log\example.example" logLevel="7" targetType="DataOrPathFile">
     <analysisManagers>
       <analysisManager name="AnalysisManagerSegmentCounter"
className="ini.nereda.tutorial.analysis.AnalysisManagerSegmentCounter">
         <subAnalyses>
           <subAnalysis name="AnalysisSegmentCounter" active="true"></subAnalysis>
         </subAnalyses>
         <targets>
           <target className="ini.nereda.NeuronInf" />
         </targets>
         <plotters defaultPlottersActive="true">
         </plotters>
         <custom>
           <params>
           </params>
         </custom>
       </analysisManager>
     </analysisManagers>
     <viewManagers>
       <viewManager name="BrainViewManagerDendrogram"
className="ini.nereda.viewManager.BrainViewManagerDendrogram">
         <targets>
           <target className="ini.nereda.NeuronInf" />
         </targets>
         <custom>
         </custom>
       </viewManager>
     </viewManagers>
```

```
      <externalDataCreation>
      </externalDataCreation>
    </job>
  </jobs>
</nereda>
```

The *jobs* section contains a *job* node for each Nereda job. Within the job you have the sections: *analysisManagers*, *viewManagers* and *externalDataCreation*. In the *analysisManagers* you have an analysis manager node, which informs Nereda what analysis manager to load, to what target(s) it should be attached to, and what plotters should be run after the analysis is finished. Beside of that analysis manager specific parameters can be set in the *params* section. The same principle applies to the *viewManagers* saection which informs the Nereda job project what view should be generated, on what targets.

This specific job configuration would create an *AnalysisManagerSegmentCounter* instance, which attaches an *AnalysisSegmentCounter* object to each neuron, starts it, and saves the results to a Matlab mat-file. Subsequently the default plotters of the analysis objectare run, which visualize the data. In addition this job creates a *BrainViewManagerDendrogram* object, which creates a *BrainViewDendrogram* object, attaches it to each neuron, and shows the axonal and dendritic trees as a dendrogram. Again the results are saved automatically.

The *externalDataCreation* section is empty in this example. It could contain configuration nodes for external data handlers that are capable to create external data, e.g. pinwheel coordinates on angle maps.The config node for this would look the following: `<handler name= "ExternalDataHandlerPinwheelCoords" className = "ini.nereda.project. externalData. ExternalDataHandlerPinwheelCoords "></handler>` . If Nereda starts this job, it would before executing all other analysis or viewers, start the external data creation, which lets you select the pinwheels on the angle map. Make sure that the project file contains all required brain features and external data handlers in its registries, otherwise exceptions will be thrown.

Visitors

The visitor pattern is the fundamental technique how Nereda collects and processes data in the object tree. The pattern separates function implementation and function target into distinct classes, i.e. the visitor and the visited object (target). In this way additional functionality can be added to the framework, without actually having to change the interface of the target class, which is a particular advantage in situations where the requirements are highly dynamic, e.g. in research.

The visitor which implements *VisitorInf* can be seen as a little mobile code package that traverses automatically a given subtree and stops when it has returned to its intitial position. The framework guarantees that each tree object withing the scope is visited *twice*. The first time when the visitor travels down towards the leafs and the second time when it is returning, back to its initial position. Each Nereda object implements the *VisitedInf* interface, and is therefore a potential target for a visitor.
VisitedInf has three methods:

- void visit(VisitorInf visitor) throws Exception;
- void visitDeep(VisitorInf visitor) throws Exception;
- void visitDeepShallow(VisitorInf visitor) throws Exception;

They define the scope of the visitor. *visit* will instruct the visitor to only visit the initial target object, while ignoring the deeper levels of the subtree. *visitDeep* tells the visitor to visit each object of the subtree that is rooted in the intial object (the one the visitDeep method is called on). *visitDeepShallow* is a convenience method for an intermediate behavior, where the visitor visits the intial object and its direct children.

The *VisitorInf* interface contains mainly only two methods, *execVisit(ObjectInf visitedObj)* and *execAfterVisit(ObjectInf visitedObj)* which are executed when the visitor encounters the visited object the first time (execVisit) and on its way home (execAfterVisit). The two methods allow for flexible and elegant implementation of a large number of requirements.

A simple implementation for a visitor that collects the axonal segments length could look like this:

```
package ini.nereda.visitor;
import ini.nereda.AxonalSegment;
import ini.nereda.AxonalSegmentInf;
import ini.nereda.ObjectInf;
import java.util.Vector;
public class AxonalSegmentLengths extends AbstractVisitor {
  private Vector<Double> segmentlengths = new Vector<Double>();
  @Override
  public void execVisit(ObjectInf visitedObj) throws Exception{
            if(visitedObj instanceof AxonalSegment)
            {
                      aquireinfo(visitedObj.getAsAxonalSegment());
            }
  }
            private void aquireinfo(AxonalSegmentInf seg){
            segmentlengths.add(seg.getLength());
  }
            @Override
  public Vector<Double> terminate() {
            return segmentlengths;
  }
 }
```

Analysis

An analysis class contains methods to perform data processing and saving the results as Matlab (MAT) files. The class has to implement the interface *AnalysisInf* of the *ini.nereda.data.analysis* package.

At runtime the analysis target is determined which is by default the object the analysis is attached to. A target object can hold any number and any type of analysis objects , as long as they implement AnalaysisInf. An analysis object in contrast only supports certain target object types, e.g. some do only work if they are attached to a neuron. The analysis provides the valid target types by virtue of the *getValidTargets()* method. If the target is not of a valid type, a runtime exception will be thrown. Beside of that the analysis informs the environment about its prerequisites by the *getRequiredBrainFeatures()* method, which returns a set of brain features that have to be loaded before the analysis is executed (usually done automatically by the project machinery).

Depending on the target the same analysis may produce different results, since different subsets of the whole object tree are treated. An analysis can provide default plotters that visualize the analysis output (Matlab (FIG) figures). The list of plotters is extensible.

The Nereda core project does only provide a small subset of predefined analysis classes (*ini.nereda.data.analysis* package). The user of the framework is expected to write his/her own implementations based on their needs.

A trivial example that counts segments and computes the total length by virtue of an internal visitor class could look like this:

```
package ini.nereda.tutorial.analysis;

import ini.nereda.NeuronInf;
import ini.nereda.ObjectInf;
import ini.nereda.VisitorInf;
import ini.nereda.data.analysis.AnalysisAbstract;
```

```
import ini.nereda.data.analysis.AnalysisInf;
import ini.nereda.visitor.AbstractVisitor;
import java.util.Hashtable;

import com.mathworks.toolbox.javabuilder.MWArray;
import com.mathworks.toolbox.javabuilder.MWStructArray;
public class AnalysisSegmentCounter extends AnalysisAbstract implements AnalysisInf {
         public AnalysisSegmentCounter() {
         }
         @Override
         protected void addDefaultPlotters() {
         }
         @Override
         @SuppressWarnings("unchecked")
         public Class<? extends ObjectInf>[] getValidTargets() {
                  // The valid targets
                  return new Class[] { NeuronInf.class };
         }
         public void setActiveSubAnalysis(String[] subsAsString) {
                  // We only use a default sub-analysis (see execute())
         }
         @Override
         public MWArray execute() throws Exception {
                  // The sub-analysis name. This name will appear in the final result
                  // structure
                  String subanalysisName = "NeredaTutorial1";
                  // We create a visitor that does our work (count axonal-/dendritic
                  // segments & calculate the total length(
                  VisitorInf _segmentVisitor = new VisitorSegmentCounter();
                  // The visitor performs a deep visit on the tree, rooted in _target
                  _target.visitDeep(_segmentVisitor);
                  // The number of appendices and their lengeht are saved in result.
                  double[] result = _segmentVisitor.terminate();
                  // A result structure is defined with the fields 'totalSegmentNr' and
                  // 'totalSegmentLength' is created.
                  Hashtable<String, Object> mwMap = new Hashtable<String, Object>();
                  mwMap.put("totalSegmentNr", result[0]);
                  mwMap.put("totalSegmentLength", result[1]);
                  MWStructArray mwResultStruct = MWStructArray.fromMap(mwMap);
                  // The structure is added to the analysis' result map
                  MWStructArray mwSubanalysisStruct = new MWStructArray(new int[] { 1, 1 },
                  new String[] { subanalysisName });
                  mwSubanalysisStruct.set(subanalysisName, new int[] { 1, 1 },
                  mwResultStruct);
                  _result.put(_target, mwSubanalysisStruct);
                  return mwResultStruct;
                  }
         private class VisitorSegmentCounter extends AbstractVisitor implements VisitorInf {
                  private int totalSegmentNr = 0;
                  private double totalSegmentLength = 0;
                  public VisitorSegmentCounter() {
                  }
                  @Override
                  public void execVisit(ObjectInf visitedObj) throws Exception {
                           super.execVisit(visitedObj);
                  // If it is an appendix object, increment the counter and update the
                           // total lenght
                           if (visitedObj.getAsAppendixSegment() != null) {
                                    totalSegmentNr++;
                                    totalSegmentLength +=
                                    visitedObj.getAsAppendixSegment().getLength();
                           }
                  }
                  @Override
                  public double[] terminate() {
                           // Return the two numbers in an array
                           return new double[] { totalSegmentNr, totalSegmentLength };
                  }
         }
}
```

Brain Viewer

A brain viewer is the sister class of a analysis. They both follow the same principles. But in contrast to an analysis a viewer is intended to produce visualizations of the brain or brain components. Hence rather than performing analysis steps that end up with a MAT file, it will produce a list of figures. The boundary between the two is not sharp however. An analysis with attached plotters can produce partially the same output as a viewer.

Manager classes

Analysis and brain viewer classes are accompanied by manager classes that control analysis object instantiation, setting the targets, execution of the analysis and the automatic saving of the results. The manager classes are used in the context of *Nereda jobs*, where actions have to be performed automatically, without user or programmer interaction. Which analysis or view manager is used in a given job, is configured in the Job configuration file. At runtime the configured manager is created by virtue of Java reflection. The manager subsequently creates the corresponding analysis object or viewer and controls its behavior.

The naming conventions for class names are AnalysisManager<Analysis name> and BrainViewManager<View name>.

A typical analysis manager for an analysis class with one input parameter could look like this:

```
package ini.nereda.data.analysis.manager.horizontalconnections;

import ini.nereda.data.analysis.horizontalconnections.AnalysisCBLVsLayer;
import ini.nereda.data.analysis.manager.AnalysisManagerAbstract;
import ini.nereda.exception.InvalidDataException;
import ini.nereda.project.ConfigXPathExpressions;
import ini.nereda.project.ConfigXmlHelper;
import java.io.File;
import java.io.InvalidClassException;
import javax.xml.xpath.XPathExpressionException;
import org.w3c.dom.Node;
public class AnalysisManagerCBLVsLayer extends AnalysisManagerAbstract {
  public AnalysisManagerCBLVsLayer(File outPath, Node analysisManagerNode) throws
InvalidClassException,
                    XPathExpressionException, ClassNotFoundException,
InvalidDataException {
            super(analysisManagerNode, outPath);
            String layerIdToNormalizeCBLToKey =
ConfigXPathExpressions.PARAM_LAYERID_TO_NORM_CBL_TO;
            String layerIdToNormalizeCBLTo;
            try {
                    layerIdToNormalizeCBLTo =
ConfigXmlHelper.getParamValue(analysisManagerNode, layerIdToNormalizeCBLToKey);
                    _analysis = new AnalysisCBLVsLayer(layerIdToNormalizeCBLTo);
                    init();
            } catch (XPathExpressionException e) {
                    throw e;
            }
  }
}
```
